# Defective neutrophil clearance in *JAK2^V617F^* myeloproliferative neoplasms drives myelofibrosis via immune checkpoint CD24

**DOI:** 10.1182/blood.2024027455

**Published:** 2025-08-07

**Authors:** Eman Khatib-Massalha, Christian A. Di Buduo, Agathe L. Chédeville, Ya-Hsuan Ho, Yexuan Zhu, Elodie Grockowiak, Yuki Date, Lam T. Khuat, Zijian Fang, José Quesada-Salas, Eva Carrillo Félez, Matteo Migliavacca, Isabel Montero, José A. Pérez-Simón, Alessandra Balduini, Simón Méndez-Ferrer

**Affiliations:** 1 Cambridge Stem Cell Institute, University of Cambridge, Cambridge CB2 0AW, United Kingdom; 2 Department of Haematology, University of Cambridge, Cambridge CB2 0AW, United Kingdom; 3 NHS Blood and Transplant, Cambridge CB2 0AW, United Kingdom; 4 Department of Molecular Medicine, University of Pavia, 27100 Pavia, Italy; 5 Department of Biomedical Engineering, Tufts University, Medford MA 02155, Massachusetts, USA; 6 Instituto de Biomedicina de Sevilla-IBiS ( Hospital Universitario Virgen del Rocío / CSIC/ Universidad de Sevilla), 41013 Seville, Spain; 7 Department of Medical Physiology and Biophysics, University of Seville, 41009 Seville, Spain; 8 Department of Hematology, University Hospital Virgen del Rocío, 41013 Seville, Spain

## Abstract

Myeloproliferative neoplasms (MPNs) are hematopoietic stem cell-driven malignancies marked by excessive myelopoiesis and high risk of myelofibrosis, which remains therapeutically challenging. Senescent neutrophils home daily to the bone marrow (BM) to be cleared by macrophages. This avoids their accumulation, which can increase the risk of chronic inflammation or oncogenesis. Neutrophils carrying the most common oncogenic MPN driver (*JAK2^V617F^*) are protected from apoptosis, which may prolong their lifespan and enhance their pro-inflammatory activity. On the other hand, abnormal interactions of neutrophils with megakaryocytes (“emperipolesis”) have been associated with BM fibrosis in disparate hematological disorders, including MPN and grey platelet syndrome; however, the underlying pathophysiology remains unclear.

We investigated neutrophil homeostasis and cellular interactions in MPN. We found that senescent neutrophils evade homeostatic clearance and accumulate in *JAK2^V617F^* MPN, but not in MPN caused by the second most prevalent mutations affecting *Calreticulin* (*CALR*) gene. This is explained by GM-CSF-JAK2-STAT5-dependent upregulation of the ″don’t-eat-me″ signal CD24 in neutrophils. Mechanistically, *JAK2^V617F^* CD24^hi^ neutrophils evade efferocytosis, invade megakaryocytes and increase active TGF-β. Collectively, *JAK2^V617F^* neutrophil-megakaryocyte interactions promote platelet production in a humanized bioreactor and myelofibrosis in mouse models. Notably, chronic antibody blockade or genetic loss of CD24 restores clearance of senescent neutrophils, reduces emperipolesis and active TGF-β. Consequently, CD24 blockade improves thrombocytosis and prevents myelofibrosis in MPN mice. Taken together, these findings reveals defective neutrophil clearance as a cause of pathogenic microenvironmental interactions of inflammatory neutrophils with megakaryocytes, associated with myelofibrosis in MPN. Our study postulate CD24 as a candidate innate immune checkpoint in MPN.

## Introduction

While neutrophils are the most abundant leukocytes and essential components of the innate immune response, upon recruitment to the tumor microenvironment they can be functionally reprogrammed to promote cancer growth^1,2^. Therefore, the homeostatic clearance of senescent neutrophils (efferocytosis) is crucial to prevent chronic inflammation, cytotoxic tissue damage, autoimmune or pro-tumorigenic processes^3^. MPNs are clonal hematopoietic disorders characterized by excessive production of myeloid cells driving chronic inflammation, which promotes difficult-to-treat BM scarring (myelofibrosis)^4–6^.

Circulating HSCs and leukocytes home daily to the BM^6^, where senescent/apoptotic neutrophils are cleared physiologically by resident macrophages^7–9^. However, neutrophil efferocytosis decreases during aging^10^, which is associated with a higher risk of MPN. Furthermore, neutrophils carrying the most common MPN mutation (*JAK2^V617F^*) are overproduced and protected from apoptosis^11–15^. *JAK2^V617F^* neutrophils release inflammatory cytokines and proteolytic enzymes, trigger the formation of neutrophils extracellular traps (NETs), aggregate with platelets and activated endothelium, favoring coagulation and increasing the risk of thrombosis^16–20^. On the other hand, abnormal interactions of neutrophils with megakaryocytes (“emperipolesis”) have been associated with BM fibrosis in disparate hematological disorders, including MPN^21^ and grey platelet syndrome^22^; however, the pathophysiological implications of emperipolesis remain unclear^23^. Here, we describe neutrophil homeostasis and its cellular interactions relevant to the pathophysiology of MPN.

## Methods

### Human studies

All centers had appropriate research and ethical approval. Human peripheral blood samples were obtained from healthy subjects and patients with MPNs after informed consent. Samples were derived from the Cambridge Biobank and Addenbrooke’s Hospital (UK), the Andalusian Public Health System Biobank at Virgen del Rocío Hospital (Spain), and the I.R.C.C.S Policlinico San Matteo Foundation of Pavia (Italy). All research involving human material was approved by Ethical Committees and conducted adhering to the Declaration of Helsinki.

### Experimental details

Cell extraction and culture^24^, flow cytometry and cell sorting^25^ and 3D cell culture in the silk BM model^26,27^ have been described previously. Further details about these and mouse models, treatments, functional assays, statistics and reproducibility are provided in the ‘supplemental materials and methods’ section.

## Results

### Senescent neutrophils accumulate in MPN BM due to defective clearance

**Figure 1:**
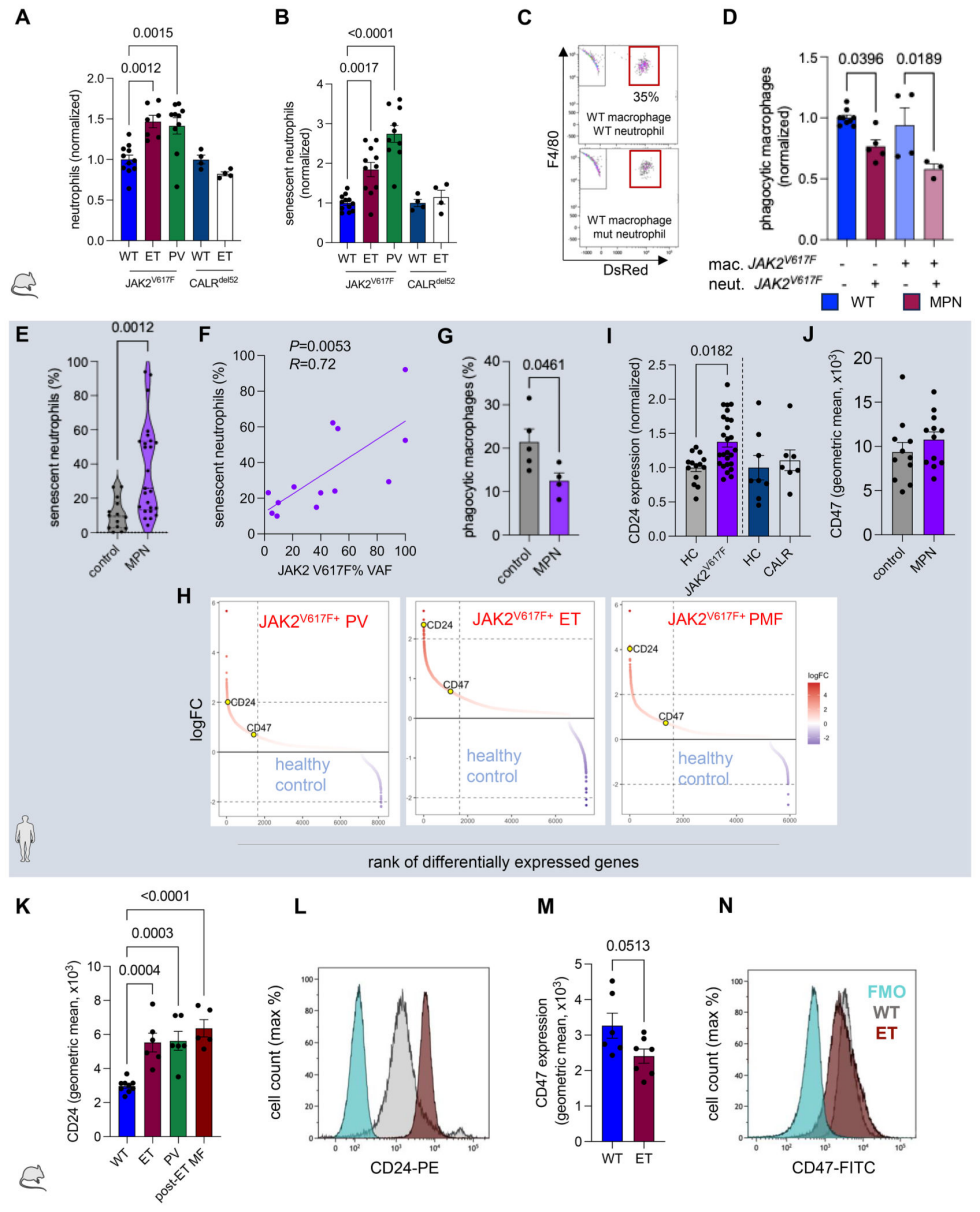
Deficient clearance of senescent neutrophils in MPN, caused by CD24 “don’t-eat-me” signal. (A-B) Normalized frequency of BM neutrophils (A; n=4-11 mice per group) and senescent neutrophils (B; n=4-13 mice per group) in WT mice and MPN mouse models of essential thrombocythemia (ET) or polycythemia vera (PV) carrying *JAK2^V617F^* mutation, ET-like mice carrying the second most common mutation (*CALR^del52^*) and their WT controls. Cells were harvested between 8-10 am. (C-D) Flow cytometry plots (C) and normalized frequency (D) of WT or MPN macrophages phagocytosing neutrophils from WT or MPN (ET-like or PV-like) mice (n=3-9). Note that *JAK2^V617F^* in neutrophils (but not macrophages) reduces efferocytosis. (E) Frequency of circulating senescent neutrophils from MPN patients (n=25) and healthy controls (n=14). (F) Correlation of *JAK2^V617F^* variant allele frequency (VAF) in circulating granulocytes and the frequency of senescent neutrophils in the peripheral blood of MPN patients (n=20). (G) Frequency of human phagocytic macrophages in co-culture with granulocytes from MPN patients or healthy controls (n=4-5 per group). (H) Supervised analyses of human blood granulocytes (PV, n=28; ET, n=47; myelofibrosis, MF, n=18) and healthy controls (n=11)^30^. (I-J) CD24 (I) or CD47 (J) protein expression in blood neutrophils from MPN patients carrying *JAK2^V617F^* (I, n=26; J, n=12) or *CALR^del52^* mutation (I, n=7) and healthy controls (I, n=8-14; J, n=11). (K) BM CD24 protein expression in senescent neutrophils from WT (n=9), ET-like (n=6), PV-like (n=6) or post-ET myelofibrosis (MF; n=5) mice. ET or PV mice were analyzed at early stage of disease (age 13-16w), post-ET MF at late stage (age 20-24w). Cells were harvested between 8-10 am. (L) Flow cytometry histogram showing geometric mean of CD24 expression in BM senescent neutrophils from ET-like or WT mice. (M) BM CD47 protein expression in senescent neutrophils from WT (n=6) or ET-like (n=7) mice. Cells were harvested between 8-10 am. (N) Flow cytometry histogram plot showing geometric mean of CD47 expression in BM senescent neutrophils from ET-like or WT mice. (A-B, D, E, G, I-K,M) Data are mean±SEM. Each dot is a mouse or individual. (A-B, D, I, K) One-way ANOVA and pairwise comparisons. (E, G, J, M) Student’s two-tailed unpaired *t* test. (F) Pearson’s correlation.

First, we measured senescent neutrophils in the peripheral blood and BM of WT/MPN mice. Senescent neutrophils were characterized as Ly6G^hi^/CD62L^lo^ cells, which showed high expression of the homing receptor CXCR4^8^ (supplemental Figure 1A). MPN senescent neutrophils displayed high expression of CXCR4, CD11b and CD45, and reduced forward scattering properties, which reflect changes during aging (supplemental Figure 1B-F). As previously described^8^, senescent neutrophils were more abundant in circulation during the day, whereas they peaked at night in the BM, where they home to be cleared by macrophages^7^ (supplemental Figure 1G-H). To study MPN neutrophil efferocytosis, we used mice carrying *JAK2^V617F^* mutation driven by *Mx1-Cre* or *Vav1-Cre*, which respectively develop MPN resembling polycythemia vera (PV) or essential thrombocythemia (ET)^28^, and ET-like mice carrying the second most common mutation (*CALR^del52^*)^29^. During daytime, BM neutrophils were 20%-increased and senescent neutrophils were 2-3-fold higher in *JAK2^V617F^* MPN, but not in *CALR^del52+^* MPN mice (Figure 1A-B), suggesting that *JAK2^V617F^* leads to the accrual of senescent neutrophils.

We next asked whether the accumulation of senescent neutrophils in MPN BM is solely caused by their overproduction, or also due to altered clearance (efferocytosis). BM cells were differentiated into macrophages and co-cultured with labelled BM neutrophils aged *ex vivo* (supplemental Figure 1I-J). Senescent neutrophil accumulation in MPN BM was explained by decreased efferocytosis; interestingly, *JAK2^V617F^* mutation in macrophages did not affect their phagocytic function, suggesting a dominant role of *JAK2^V617F^* in neutrophils (Figure 1C-D). These findings were validated in human MPN (patients’ characteristics are detailed in supplemental Table 1): circulating senescent neutrophils were high in *JAK2^V617F^* MPN patients (Figure 1E; supplemental Figure 1K). The frequency of senescent neutrophils directly correlated with *JAK2^V617F^* variant allele frequency in granulocytes (Figure 1F), suggesting that pathogenic JAK-STAT signaling causes the accumulation of senescent neutrophils. Indeed, the *JAK2^V617F^* mutation reduced the efferocytosis of human senescent neutrophils (Figure 1G), validating our previous findings.

### Defective clearance of MPN neutrophils is associated with the ″don’t-eat-me″ signal CD24

The homeostatic recognition and efferocytosis of neutrophils by macrophages rely on ″eat-me″ and ″don’t-eat-me″ signals^9^. Supervised analyses of human granulocyte microarray datasets from MPN patients and healthy donors^30^ showed moderate expression of the ″don’t-eat-me″ signal CD47 in MPN, and comparatively higher levels of CD24 (Figure 1H; Supplementary Table 2), which reportedly functions as a ″don’t-eat-me″ signal in solid tumors^31^. Higher mRNA content was matched by protein abundance, since CD24 (not CD47) protein expression was high in blood neutrophils from *JAK2^V617F^* MPN patients (but not in *CALR*-mutated MPN patients), compared with healthy controls (Figure 1I-J; supplemental Figure 2A).

High CD24 (not CD47) protein expression was detected in BM neutrophils from mouse models of PV, ET or secondary (post-ET) myelofibrosis (Figure 1K-N). In these models, CD24 expression was notoriously higher in neutrophils compared with other immune cells and hematopoietic stem and progenitor cells (supplemental Figure 2B-C). This was validated in human MPN, where CD24 expression was negligible in HSPCs, compared with neutrophils (supplemental Figure 2D-E).

The CD24 ligand SIGLEC-10/Siglec-G mediates CD24-dependent immune evasion in solid tumors^31^. Therefore, we measured the expression of Siglec-G in mouse BM macrophages and found unchanged protein levels in MPN (supplemental Figure 2F). Thus, CD24 was prioritized as a candidate target.

### Blockade of CD24 improves neutrophil clearance in MPN

**Figure 2:**
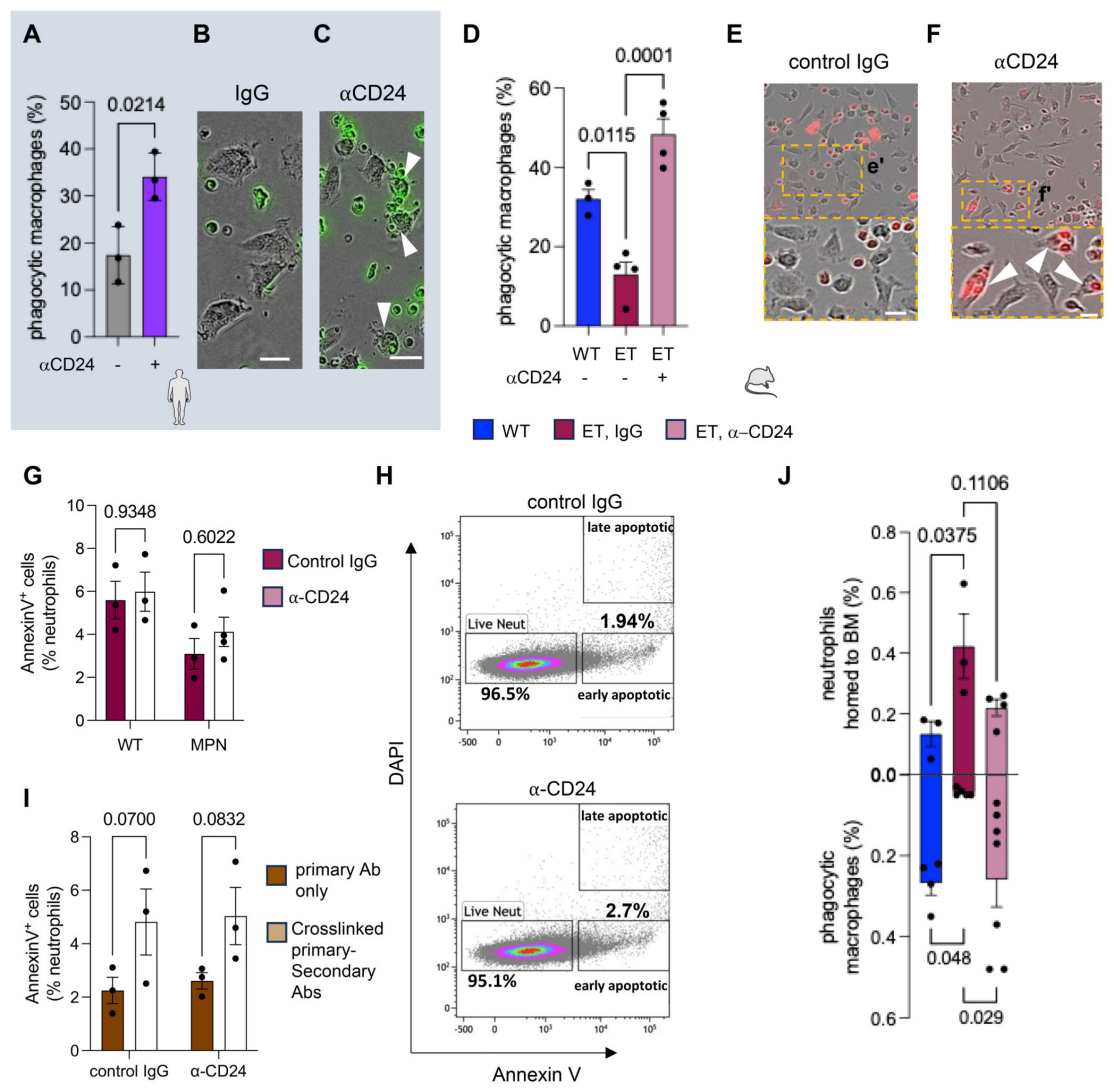
Blockade of CD24 improves neutrophil efferocytosis in MPN mice. (A-C) Frequency (A) and combined fluorescence and bright field microscopy (B-C) of human phagocytic macrophages in co-culture with labelled MPN granulocytes (green), treated 3h with anti-CD24 (clone SN3) or isotype control (mouse IgG1) antibodies (n=3). Arrowheads depict phagocytic macrophages. Scale bar, 10μm. (D-F) Frequency (D) and combined fluorescence and bright field microscopy (E-F) of WT macrophages phagocytosing β-actin-DsRed^+^ neutrophils (red) from *β-actin-DsRed* control mice (n=3) or ET-like mice (compound *β-actin-DsRed;Vav-Cre;JAK2^V617F^* mice; n=4) treated 3h with anti-CD24 antibody (clone M1/69) or isotype control (rat IgG2b). Arrowheads depict phagocytic macrophages. Scale bar, 10μm. (G-H) Frequency (G) and flow cytometry plots (H) of AnnexinV^+^ apoptotic WT or MPN neutrophils after 3h culture with anti-CD24 antibody or control IgG (n=3-4 mice per group). (I) Frequency of AnnexinV^+^ apoptotic MPN neutrophils after 22h culture with primary anti-CD24 antibody, isotype control (IC; control IgG) or with crosslinked primary-secondary antibodies (n=3 mice per group). Primary and secondary antibodies pre-incubated before addition to cells at a 2 : 1 ratio to ensure efficient cross-linking of primary antibody and to ensure no excess secondary antibody present in the culture. (J) Frequency of WT (n=4) or ET-like (n=5-7) neutrophils (top), mirroring macrophages phagocytosing WT (n=3) or ET neutrophils (n=3-4), 24h after adoptive transfer of the same number of *JAK2^V617F^*-mutated/unmutated senescent neutrophils into WT mice treated with anti-CD24 blocking antibody or control IgG (see supplementary Figure 2J for experimental design). Cells were harvested between 8-10 am. (A, D, G, I, J) Data are means±SEM. Each dot is a mouse or individual. (A) Student’s two-tailed unpaired *t* test. (D) One-way ANOVA and pairwise comparisons. (G, I-J) Two-way ANOVA and pairwise comparisons.

To investigate the possible ″don’t-eat-me″ function of CD24 in human MPN neutrophils, *in vitro* efferocytosis was tested using anti-CD24 blocking antibody or control IgG. Blocking CD24 increased MPN neutrophil efferocytosis by 20% (Figure 2A-C; supplemental Figure 2G-H), uncovering CD24 function in MPN neutrophils. Similar results were observed in MPN mice, as CD24 blockade restored normal efferocytosis in MPN neutrophils (Figure 2D-F), without significantly affecting the minimal frequency of apoptotic neutrophils (Figure 2G-H). Since antibodies may broadly trigger macrophage phagocytosis through Fc-dependent cellular cytotoxicity^32^, CD47 blockade was tested for comparison and it did not elicit phagocytosis (supplemental Figure 2I), demonstrating the specific role of CD24.

In hematopoietic cells, antibody-mediated CD24 crosslinking can induce apoptosis^33,34^, which is prevented by *JAK2^V617F^* ^30^. We tested CD24 crosslinking and found minimal (~5%) neutrophil apoptosis, which was similarly observed using control IgG (Figure 2I). These results support the role of CD24 as a ″don’t-eat-me″ signal in MPN neutrophils.

We validated these findings *in vivo* through the adoptive transfer of the same number of WT or *JAK2^V617F^*-mutated neutrophils into WT mice (supplemental Figure 2J). After 24h, transferred mutant neutrophils accumulated in the BM (3-fold-increased), which was mirrored by reduced frequency of BM phagocytic macrophages; however, CD24 blockade restored normal neutrophil frequency and homeostatic clearance (Figure 2J). These results confirm CD24 function as a ″don’t-eat-me″ signal for *JAK2^V617F^*-mutated neutrophils *in vivo*.

### GM-CSF induces CD24 expression in senescent neutrophils via JAK2-STAT5 signaling

**Figure 3:**
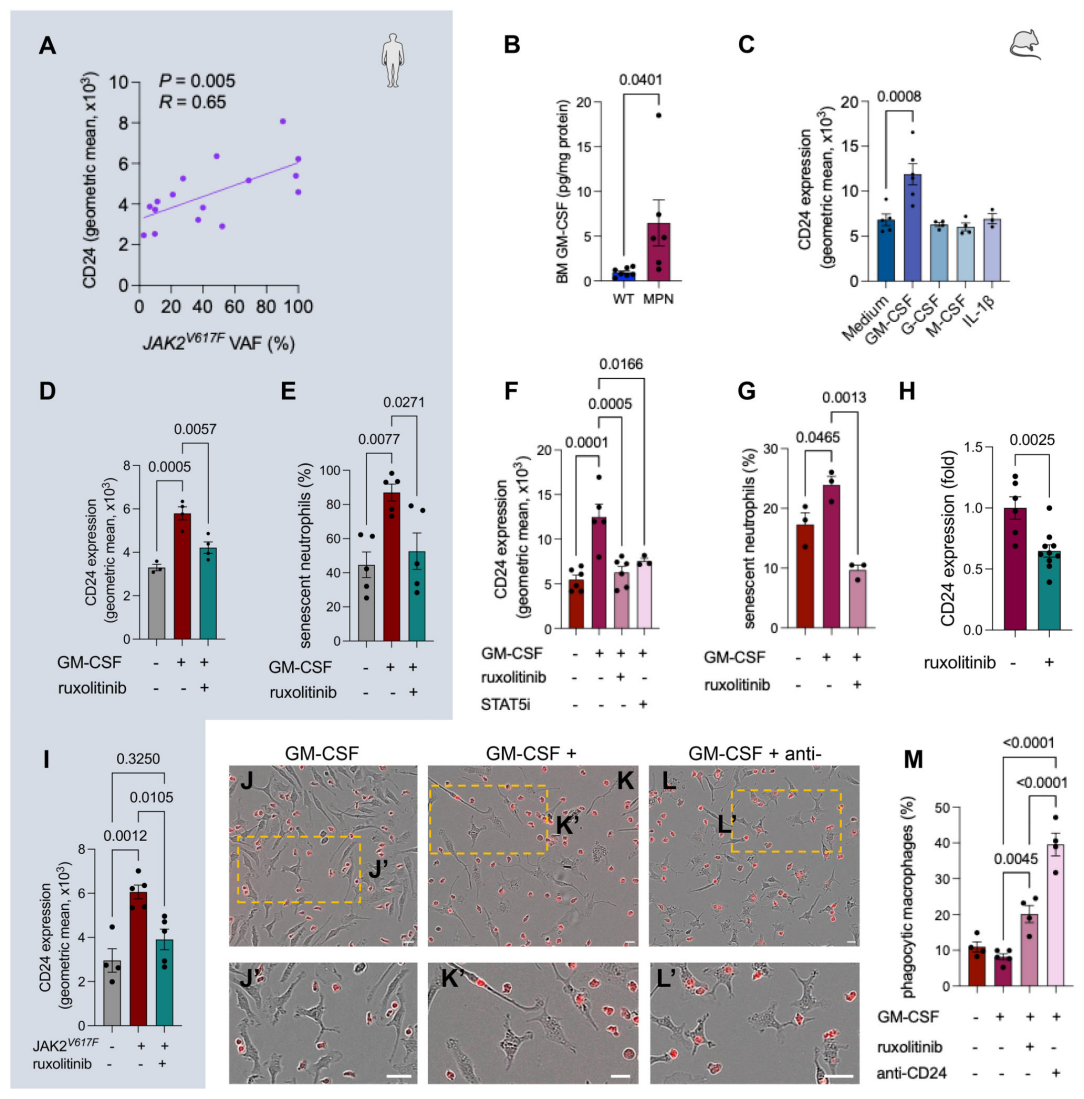
GM-CSF induces CD24 expression in senescent neutrophils via JAK2-STAT5 signaling. (A) Correlation of *JAK2^V617F^* variant allele frequency (VAF) in circulating granulocytes and CD24 protein expression in peripheral blood neutrophils from MPN patients (n=16). (B) GM-CSF concentration in central BM of WT or MPN mice (n=6-7 per group). (C) CD24 expression in senescent neutrophils from MPN mice after 16h culture with GM-CSF (n=6), G-CSF (n=4), M-CSF (n=4), IL-1 β (n=3) or medium only (n=5). (D-E) CD24 expression (D) and frequency of senescent neutrophils (E) from MPN patients after 16h culture with GM-CSF or vehicle, in presence/absence of JAK inhibitor ruxolitinib (n=4-5 per group). (F) CD24 expression in senescent neutrophils from ET-like mice after 16h culture with GM-CSF (n=5), ruxolitinib (n=6), STAT5 inhibitor (AC-4-130, n=3) or vehicle (n=6). (G) Frequency of senescent neutrophils from ET-like mice after 16h culture with GM-CSF, ruxolitinib or vehicle (n=3 per group). (H) CD24 expression (fold change) in BM senescent neutrophils from ET-like mice treated for 2.5w with ruxolitinib (twice weekly; n=10), compared with control vehicle (n=6). (I) CD24 protein expression in blood neutrophils from MPN patients receving JAK inhibitor ruxolitinib treatment compared with healthy control or MPN patients treated with other drugs (n=4-5 per group). (J-M) Combined immunofluorescence and bright field microscopy (red, J-L) and quantification (M) of phagocytic macrophages co-cultured with DsRed^+^ neutrophils from ET-like mice pre-treated with GM-CSF, alone or in combination with ruxolitinib or anti-CD24 blocking antibody (clone M1/69) (n=4-5). Scale bar, 10μm. (B-I, M) Data are means±SEM. Each dot is a mouse or individual. (A) Pearson’s correlation. (C-G, I, M) One-way ANOVA and pairwise comparisons. (B, H) Student’s two-tailed unpaired *t* test.

Next, we investigated the cause of CD24 upregulation in MPN neutrophils. CD24 expression correlated with *JAK2^V617F^* variant allele frequency in human granulocytes (Figure 3A), suggesting that pathogenic JAK-STAT signaling induces CD24 expression. Candidate JAK-STAT cytokines that are overproduced in MPN^35–37^ and activate the JAK-STAT pathway in neutrophils were tested. Granulocyte-macrophage colony-stimulating factor (GM-CSF), which is increased in human MPN^38,39^ and murine MPN BM (Figure 3B), doubled CD24 expression in mouse MPN neutrophils; this was not the case for other JAK-STAT-dependent (G-CSF) or -independent (M-CSF, interleukin-1β) cytokines (Figure 3C).

GM-CSF can delay neutrophil apoptosis and upregulate CD24 expression during sepsis^33,40^, suggesting a similar effect in MPN neutrophils via JAK-STAT signaling. We found increased GM-CSF receptor (*CFS2RA*) mRNA expression in human MPN granulocytes compared with healthy controls (supplemental Figure 2K). *In vitro*, GM-CSF treatment induced CD24 expression in human MPN neutrophils—this was blocked by the JAK1/2 inhibitor ruxolitinib (Figure 3D). Furthermore, GM-CSF increased the frequency of human or mouse MPN senescent neutrophils, which was similarly prevented by ruxolitinib (Figure 3E, G).

We investigated the STAT proteins regulating CD24 expression downstream of JAK2. *In vitro*, GM-CSF treatment induced CD24 expression in mouse MPN senescent neutrophils; this was prevented by STAT5 (not STAT3) inhibitor (blocking signaling downstream of GM-CSF receptor)^41^, phenocopying the effect of ruxolitinib (Figure 3F; supplemental Figure 2L). *In vivo*, ruxolitinib treatment reduced CD24 expression in *JAK2^V617F^* senescent neutrophils (Figure 3H). In line with these findings, CD24 expression was lower in neutrophils from ruxolitinib-treated (compared with hydroxycarbamide-treated) MPN patients (Figure 3I).

To confirm the requirement of STAT5 for GM-CSF-induced CD24 expression, neutrophils were isolated from conditional knockout (cKO) mice lacking STAT5 in the hematopoietic system. In WT neutrophils, GM-CSF doubled CD24 (not CD47) expression to a much larger extent than in neutrophils from STAT5 cKO mice (supplemental Figure 2M-O). Together, these results suggest that increased GM-CSF in MPN induces CD24 expression in mutated neutrophils via JAK2-STAT5 signaling.

Since the culture period required for *ex vivo* aging reduced CD24 expression in MPN neutrophils (in the absence of their native inflammatory environment), WT macrophages were co-cultured with GM-CSF-stimulated MPN senescent neutrophils to investigate the functional consequence of GM-CSF-induced CD24 upregulation. CD24 blockade was twice as efficient as ruxolitinib increasing phagocytosis (Figure 3J-M). This is consistent with ruxolitinib normalizing, but not blunting, CD24 expression in *JAK2^V617F^* neutrophils (Figure 3E-F). These results suggest that cytokines activating JAK-STAT signaling in neutrophils (i.e. GM-CSF) prevent their efferocytosis in MPN through CD24 upregulation.

### *JAK2^V617F^* is necessary, but not sufficient, to decrease neutrophil clearance

The effect of GM-CSF (see Figure 3D,G) suggested the possibility that the *JAK2^V617F^* mutation alone might not be sufficient to induce CD24 expression and reduce efferocytosis in MPN neutrophils. To address this question, we used mice carrying *JAK2^V617F^* in myeloid cells, but not in HSCs (*LysM-Cre;JAK2^V617F^* mice); these mice develop erythrocytosis, but not overt MPN or myelofibrosis^42^, consistent with lower levels of inflammatory cytokines, including GM-CSF (supplemental Figure 3A-E). We found normal frequencies of total or senescent BM neutrophils in *LysM-Cre;JAK2^V617F^* mice (supplemental Figure 3F-G). Furthermore, their CD24 expression was unchanged, but was doubled after *in vitro* treatment with GM-CSF (supplemental Figure 3H-I), arguing for the need of inflammatory cytokines activating JAK-STAT signaling in neutrophils, besides the *JAK2^V617F^* mutation.

### Pathogenic interactions of neutrophils evading efferocytosis with megakaryocytes in MPN

**Figure 4:**
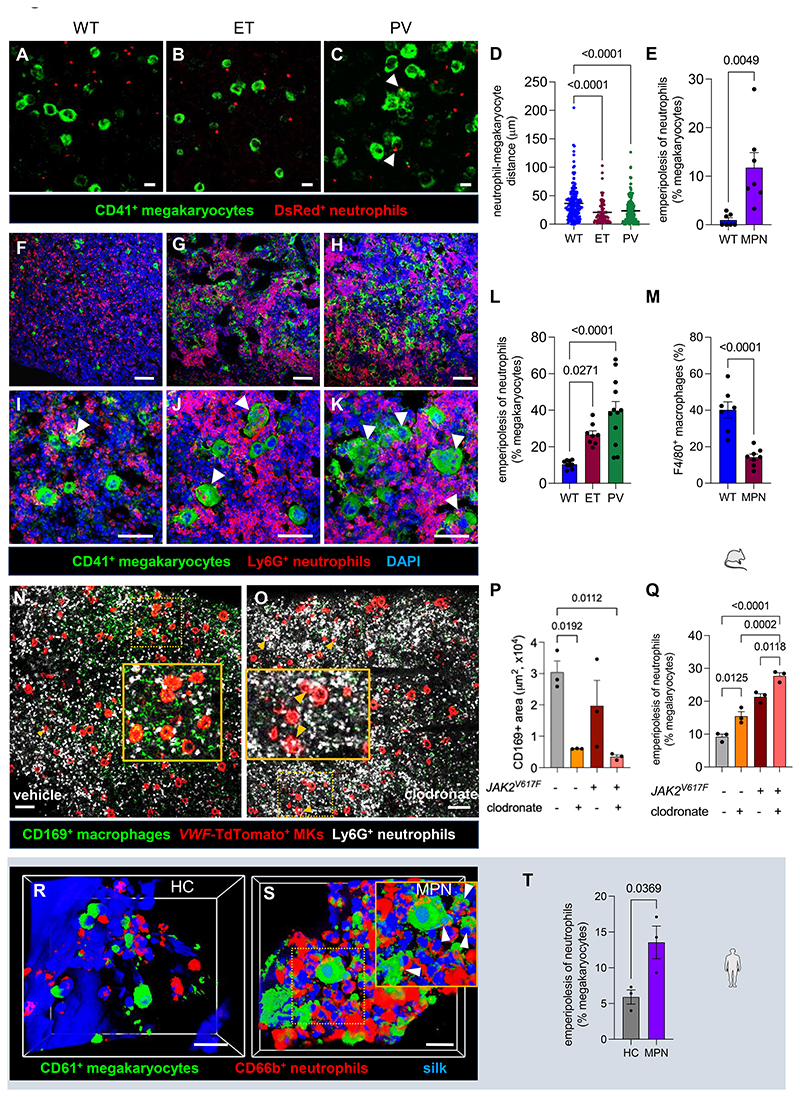
Pathogenic interactions of immune-evaded neutrophils with megakaryocytes in MPN. (A-C) Immunofluorescence of CD41^+^ megakaryocytes (FITC; green) and DsRed^+^ neutrophils (red) isolated from control mice or MPN mice, 24h after adoptive transfer into WT recipients (see supplementary Figure 2J for experimental design). ET, essential thrombocythemia. PV, polycythemia vera. Arrowheads depict cell-cell contact. Scale bar, 10μm. (D) Minimal distance between megakaryocytes and DsRed^+^ neutrophils. Each dot is a pair (WT, n=170; ET, n=82; PV, n=192). (E) Frequency of megakaryocytes manifesting emperipolesis of DsRed^+^ neutrophils (WT, n=7; MPN, n=7). (F-L) Immunofluorescence of CD41^+^ megakaryocytes (FITC; green) and Ly6G^+^ neutrophils (AF647; red) (F-K) and (L) frequency of emperipolesis (arrowheads) in the BM of WT (n=9), ET-like (n=8) or PV-like (n=12) mice. Nuclei were counterstained with DAPI (blue). Scale bar, 100μm (F-H), 50μm (I-K). (M) Frequency of CD11b^+^ F4/80^+^ macrophages among BM Ly6G^-^ cells from WT mice (n=7) or ET-like mice (n=8). (N-Q) Immunofluorescence (N-O) and frequency (P-Q) of CD169^+^ macrophages (P; green) or *VWF*-TdTomato^+^ megakaryocytes (red) showing emperipolesis (Q; arrowheads) of neutrophils (white) 24h after treatment with clodronate liposomes, to deplete macrophages, or vehicle (n=3 per group). Scale bar, 100μm. (R-T) Co-culture of megakaryocytes derived from healthy donors with human healthy control (HC) or MPN (primary myelofibrosis carrying *JAK2^V617F^*) neutrophils in a 3D silk-based BM model. (R-S) Representative examples of CD61^+^ human megakaryocytes (green) closely interacting with CD66b^+^ human neutrophils (R-S, red) and (T) frequency of emperipolesis (n=3). Scale bar, 25μm (R), 20μm (S). (D-E, L-M, P-Q, T) Data are mean±SEM. Each dot is a mouse or individual. (D, L, P-Q) One-way ANOVA and pairwise comparisons. (E, M, T) Student’s two-tailed unpaired *t* test.

We next asked whether cumulative senescent neutrophils in MPN might lead to abnormal BM microenvironmental interactions. For that, we adoptively transferred the same number of WT or *JAK2^V617F^* senescent neutrophils genetically labelled with β-actin-DsRed, to facilitate cell tracking (see supplemental Figure 2J). 24h after transfer into WT recipients, *JAK2^V617F^* neutrophils were found close to megakaryocytes (Figure 4A-D), suggesting increased interactions between senescent neutrophils and megakaryocytes in MPN.

Consistent with previous studies of emperipolesis in a different MPN model^43^, we found that *JAK2^V617F^* (but very rarely WT) senescent neutrophils undergo emperipolesis in megakaryocytes (Figure 4E). A high frequency of megakaryocyte emperipolesis of mutated neutrophils^21^ was observed in *JAK2^V617F^* MPN mice and correlated with overall disease burden, which is known to be higher for PV, compared with ET (Figure 4F-L and supplemental Figure 4A-B).

To investigate megakaryocyte-neutrophil interactions, we co-cultured BM megakaryocytes with neutrophils freshly isolated and aged *ex vivo*. Doubled frequency of emperipolesis was observed in megakaryocytes co-cultured with *JAK2^V617F^* neutrophils, compared with WT neutrophils (supplemental Figure 4C-D). We sorted young (CD62L^hi^) and senescent (CD62L^low^) neutrophils from β-actin-DsRed MPN mice and co-cultured them with megakaryocytes genetically labelled with enhanced green fluorescent protein expressed under the regulatory elements of Von Willebrand factor (*VWF-eGFP* mice)^44^ (supplemental Figure 4E). Senescent neutrophils underwent emperipolesis more frequently than young neutrophils (supplemental Figure 4F-G). Together, these findings suggest that senescent neutrophils undergo emperipolesis when they evade efferocytosis and encounter megakaryocytes, after homing back to the BM^45^.

Because BM neutrophils are phagocytosed by macrophages, we measured macrophage numbers and found them reduced in MPN mice (Figure 4M), possibly contributing to reduced efferocytosis, leading to increased emperipolesis. Supporting this possibility, emperipolesis of neutrophils increased in mice treated with clodronate liposomes to deplete phagocytic macrophages (Figure 4N-Q), suggesting that defective neutrophil efferocytosis by BM macrophages evokes neutrophil emperipolesis in megakaryocytes.

To validate these findings in a humanized system, we leveraged on a functionalized customizable perfused silk-based 3D BM model, which enables terminal maturation of human megakaryocytes under constant perfusion mimicking blood flow^46^. First, megakaryocytes derived from human CD34^+^ HSPCs were seeded in the bioscaffold (supplemental Figure 5A). Addition of human neutrophils triggered their emperipolesis in megakaryocytes, more prominently with *JAK2^V617F^* neutrophils (Figure 4R-T; supplemental Figure 5B). These results confirm our previous observations and validate the humanized system to investigate neutrophil-megakaryocyte interactions.

### Megakaryocyte emperipolesis of neutrophils increases platelet release in MPN

**Figure 5:**
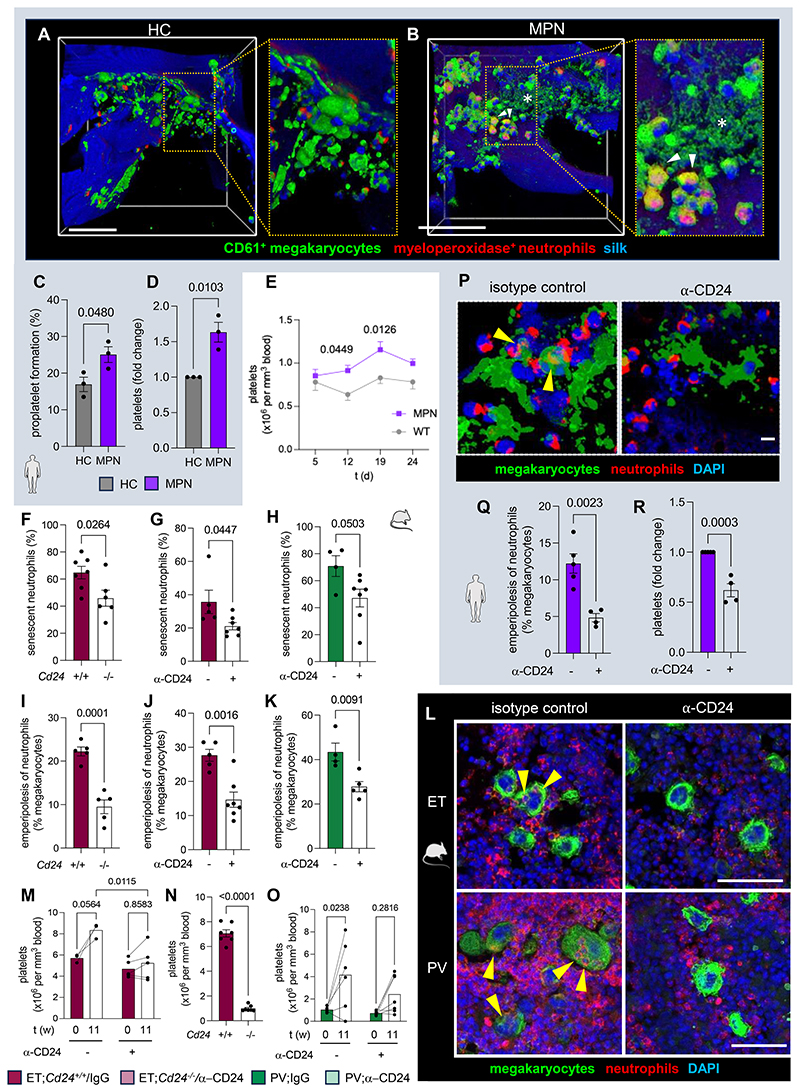
CD24 deletion or blockade restores normal clearance of senescent neutrophils, prevents emperipolesis and improves thrombocytosis in MPN. (A-B) Immunofluorescence of human CD61^+^ megakaryocytes (green) and myeloperoxidase^+^ neutrophils (red) from (A) healthy controls (HCs) or (B) MPN patients. Scale bar, 100 μm. (C-D) Quantification of (C) proplatelet formation and (D) platelet release in the 3D model under perfusion (n=3). HC, healthy control. MPN, myeloproliferative neoplasms. (E) Circulating platelet counts after repeated adoptive transfer of same number of WT or *JAK2^V617F^* senescent neutrophils into WT mice (n=9 per group). (G, J, L, M) Therapeutic effects of CD24 blockade in mouse model of essential thrombocythemia (ET). WT mice were lethally irradiated, transplanted with BM cells from ET-like mice and treated 9-10w after transplantation with anti-CD24 blocking antibody (clone M1/69) or isotype control (rat IgG2b), for 7w or 11w (see supplementary Figure 6C). (H, K, L, O) Therapeutic effects of CD24 blockade in mouse model of polycythemia vera (PV). WT mice were lethally irradiated, transplanted with BM cells from PV-like mice 8w after polyI:polyC induction and treated for 11w with anti-CD24 blocking antibody (clone M1/69) or isotype control (rat IgG2b), starting 10w after transplantation (see supplementary Figure 6C). (F, I, N) Genetic deletion of CD24 in mouse model of essential thrombocythemia (ET). WT mice were lethally irradiated, transplanted with BM cells from compound *Vav-Cre;JAK2^V617F^ Cd24^-/-^* mice or control *Vav-Cre;JAK2^V617F^* mice and were analyzed 18w after transplantation (see supplementary Figure 6B). (P-R) Co-culture of megakaryocytes derived from healthy donors with *JAK2^V617F^* neutrophils treated with anti-CD24 antibody (clone SN3) or isotype control (mouse IgG1), in a 3D silk-based BM model. (F-H) Frequency of BM senescent neutrophils (n=4-7). (I-L) Immunofluorescence of (L) CD41^+^ megakaryocytes (green) and Ly6G^+^ neutrophils (red) (ET, top; PV, bottom) and (I-K) quantification of megakaryocyte emperipolesis of neutrophils (n=4-7). Arrowheads depict emperipolesis. Scale bar, 50μm. (P-Q) Immunofluorescence of (P) CD61^+^ (human) megakaryocytes (green) and CD66b^+^ (human) neutrophils (red) and (Q) quantification of megakaryocyte emperipolesis of neutrophils (n=4-5). Arrowheads depict emperipolesis. Scale bar, 50μm. (M-O) Circulating platelets (M, O) before and 11 weeks after treatment with anti-CD24 blocking antibody (clone M1/69) or isotype control (rat IgG2b), in ET-like mice (M, n=3-5) or PV-like mice (O; n=6-7); (N) 18w after transplantation of WT mice with BM cells from compound *Vav-Cre;JAK2^V617F^ Cd24^-/-^* mice, or control *Vav-Cre;JAK2^V617F^* mice (n=6-7). (R) Platelet release in the human 3D model under perfusion (n=5). (C-K, M-O, Q-R) Data are means±SEM. Each dot is a mouse or individual. (C-D, F-K, N, Q-R) Student’s two-tailed unpaired *t* test. (E, M,O) Two-way ANOVA and pairwise comparisons.

Invasion of the demarcation membrane system of megakaryocytes by neutrophils has been suggested to promote platelet production^47,48^. We measured real-time thrombopoiesis *ex vivo* (see supplemental Figure 5A). Co-culture of megakaryocytes with human neutrophils fostered proplatelet formation and platelet release (supplemental Figure 5C-I), suggesting that megakaryocyte-neutrophil interaction stimulates thrombopoiesis. Proplatelet formation and platelet release significantly increased when megakaryocytes were co-cultured with neutrophils from MPN *JAK2^V617F^* patients, compared with healthy controls (Figure 5A-D).

To evaluate the effect of *JAK2^V617F^* neutrophils on thrombopoiesis *in vivo*, we repeatedly injected the same number of WT/mutated senescent neutrophils into WT mice (supplemental Figure 5J). Circulating platelets increased in mice receiving *JAK2^V617F^* neutrophils, but not in those that received WT neutrophils (Figure 5E; supplemental Figure 5K). These results suggest that megakaryocyte emperipolesis of mutant neutrophils, as a consequence of their defective efferocytosis, may increase thrombocytosis in MPN.

### CD24 deletion or blockade restores normal clearance of senescent neutrophils in MPN

To further investigate the role of CD24 in thrombocytosis in MPN, we intercrossed *Vav1-Cre;JAK2^V617F^* mice with *Cd24^-/-^* mice to generate MPN mice on a CD24-deficient background. As expected, BM neutrophils from *Cd24^-/-^* MPN mice lacked CD24 protein (supplemental Figure 6A). To delete CD24 in hematopoietic cells only, avoiding potential confounder effects due to widespread CD24 deletion, we generated BM chimeras using donor MPN *Cd24^-/-^* BM cells transplanted into lethally-irradiated WT recipients (supplemental Figure 6B). For comparison, BM chimeras carrying CD24-proficient *JAK2^V617F^* BM cells were treated with CD24-blocking antibody (supplemental Figure 6C). CD24 deletion or blockade did not cause hematological toxicity (supplemental Figure 6D-S). Consistent with unchanged CD24 expression in HSCs from MPN mice or patients (see supplemental Figure 2C-E), analysis of MPN mice chronically treated with CD24-blocking antibody did not affect overall hematopoietic chimerism (supplemental Figure 6N). Genetic CD24 deletion or chronic blockade reduced total and senescent neutrophils in MPN, but spared senescent neutrophils in WT mice (Figure 5F-H; supplemental Figure 7A-D). Therefore, CD24 blockade restores normal clearance of senescent neutrophils in MPN mice *in vivo*.

### CD24 deletion or blockade prevents megakaryocyte emperipolesis of neutrophils and improves thrombocytosis in MPN

Restored clearance of senescent neutrophils in MPN mice prevented their abnormal interaction with megakaryocytes, as CD24 blockade decreased megakaryocyte emperipolesis of neutrophils (Figure 5I-L); consequently, thrombocytosis developed only in MPN mice treated with control IgG, but not in those with CD24 blockade or deletion (Figure 5M-O).

CD24 can function as a ligand for P-selectin^49^, which is highly expressed by megakaryocytes in MPN^50,51^ and has been previously involved in emperipolesis^52^. Therefore, P-Selectin blockade was tested and phenocopied the effect of CD24 inhibition (supplemental Figure 7E), suggesting that CD24 actively fosters the abnormal interaction of senescent neutrophils with MPN megakaryocytes through P-Selectin binding. To directly investigate the effect of CD24 on emperipolesis and thrombocytosis in human MPN, megakaryocytes were co-cultured with human MPN neutrophils treated with anti-CD24 blocking antibody or control IgG. The emperipolesis of MPN neutrophils was reduced after CD24 blockade (Figure 5P-Q). Consequently, proplatelet formation and platelet release in the bioreactor were significantly reduced (Figure 5P, R). This effect was due to CD24 blockade in neutrophils, rather than megakaryocytes, as treatment of human megakaryocytes cultured alone did not affect proplatelet formation and platelet release (supplemental Figure 7F-G). This is consistent with normal platelet counts in *Cd24^-/-^* mice or WT mice chronically treated with anti-CD24 blocking antibody (supplemental Figure 7H-I).

Taken together, these results indicate that CD24 prevents normal efferocytosis of senescent neutrophils in MPN, increasing their abnormal interactions with megakaryocytes. Similar results from CD24 KO or blockade exclude a prominent role of antibody-dependent cellular cytotoxicity and suggest thepareutic effects of CD24 inhibition in MPN thrombocytosis.

### CD24 blockade reduces TGF-β, prevents myelofibrosis and improves osteosclerosis in MPN

**Figure 6:**
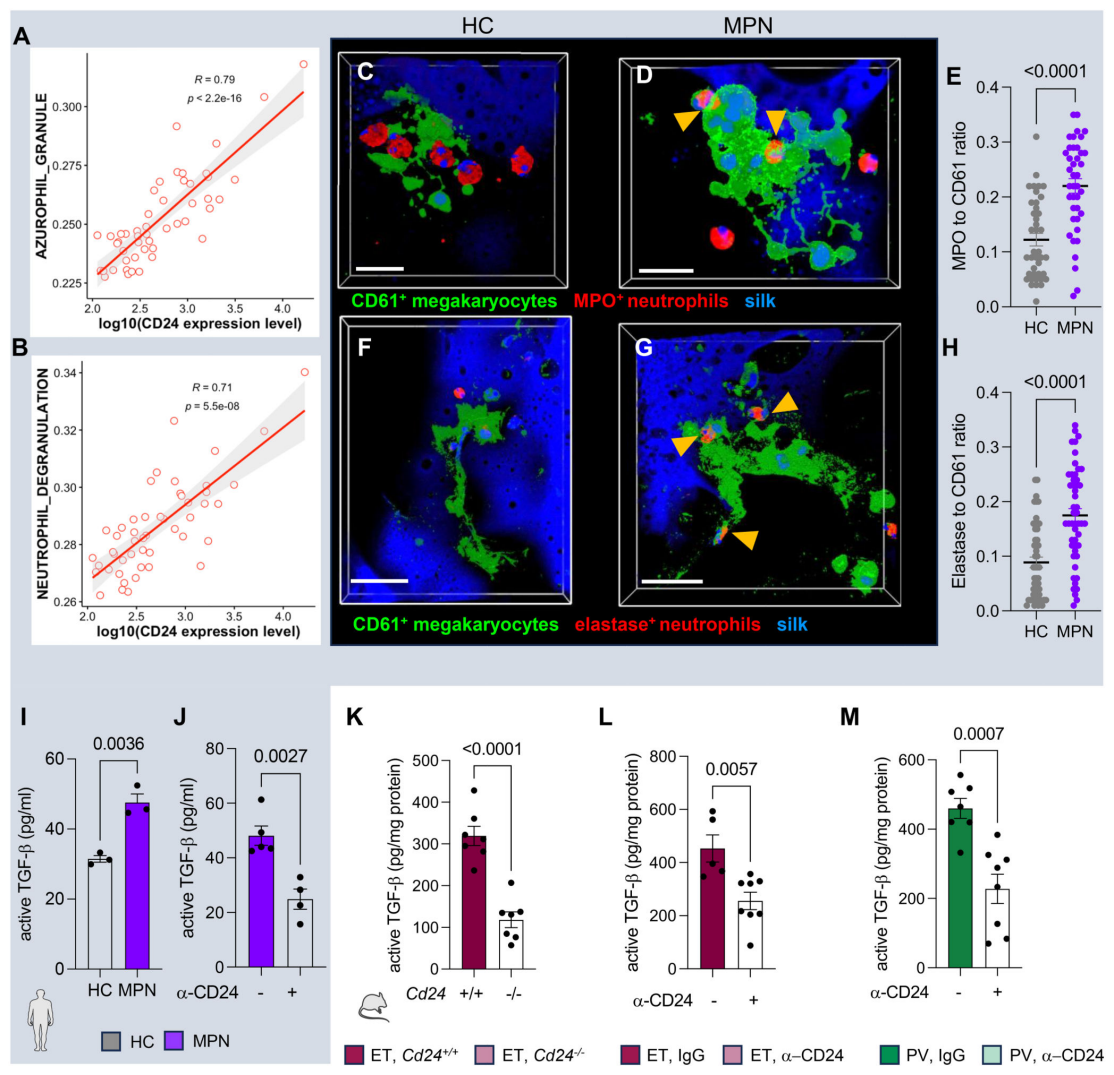
CD24 loss-of-function prevents TGF-β activation associated with degranulating neutrophils. (A-B) Supervised analysis of RNA-seq from human *JAK2^V617F^* neutrophils^56^. Correlation between CD24 expression and (A) azurophil granule or (B) neutrophil degranulation gene signatures. (C-H) Immunofluorescence (C-D, F-G) and quantification (E, H) of CD61^+^ (green) megakaryocytes, (C-D) myeloperoxidase^+^ (red) neutrophils and (F-G) elastase^+^ (red) neutrophils. Scale bar, 20μm (C-D), 50μm (F-G). (E, H) Ratio of mean fluorescence intensity (MFI) of (E) myeloperoxidase (MPO) and (H) elastase relative to CD61^+^ megakaryocytes. (I-J) Active TGF-β concentration in medium collected from co-culture of megakaryocytes with (I) neutrophils from *JAK2^V617F^* MPN patients or healthy controls (HC, n=3 per group) or (J) *JAK2^V617F^* MPN neutrophil treated with anti-CD24 antibody (clone SN3) or isotype control (mouse IgG1) (n=5 per group). (K) Active TGF-β concentration in central BM 18w after transplantation of WT mice with BM cells from compound *Vav-Cre;JAK2^V617F^ Cd24^-/-^* mice or control *Vav-Cre;JAK2^V617F^* mice (n=7). (L-M) Active TGF-β concentration in central BM of (L) ET-like mice treated for 7w with anti-CD24 blocking antibody, or control IgG (n=5-8), and (M) PV-like mice treated for 11w with anti-CD24 blocking antibody (clone M1/69) or isotype control (rat IgG2b) (n=7-8). (E, H-M) Data are means±SEM. Each dot is a mouse or individual. (E, H-M) Student’s two-tailed unpaired *t* test.

Megakaryocyte emperipolesis of neutrophils correlates with BM fibrosis development in disparate hematological disorders, such as MPN^21^ and grey platelet syndrome^22^. Myelofibrosis can arise from pro-fibrotic cytokines overproduced by megakaryocytes, like TGF-β^53^, which additionally regulates proplatelet formation^54^. TGF-β is synthesized in a latent form and requires proteolytic cleavage for activation^55^, raising the question of whether neutrophil-derived proteases contribute to this process. A supervised analysis of RNA-seq from human *JAK2^V617F^* neutrophils^56^ supported this possibility, since CD24 (not CD47) mRNA expression and gene signatures corelated with degranulation and protease-rich azurophil granules in *JAK2^V617F^* MPN neutrophils (Figure 6A-B; supplemental Figure 8A-C). Moreover, a high concentration of neutrophil-derived myeloperoxidase and elastase proteases was detected in human megakaryocytes showing emperipolesis of *JAK2^V617F^* neutrophils in the 3D bioscaffold (Figure 6C-H; supplemental Figure 8E-F).

**Figure 7:**
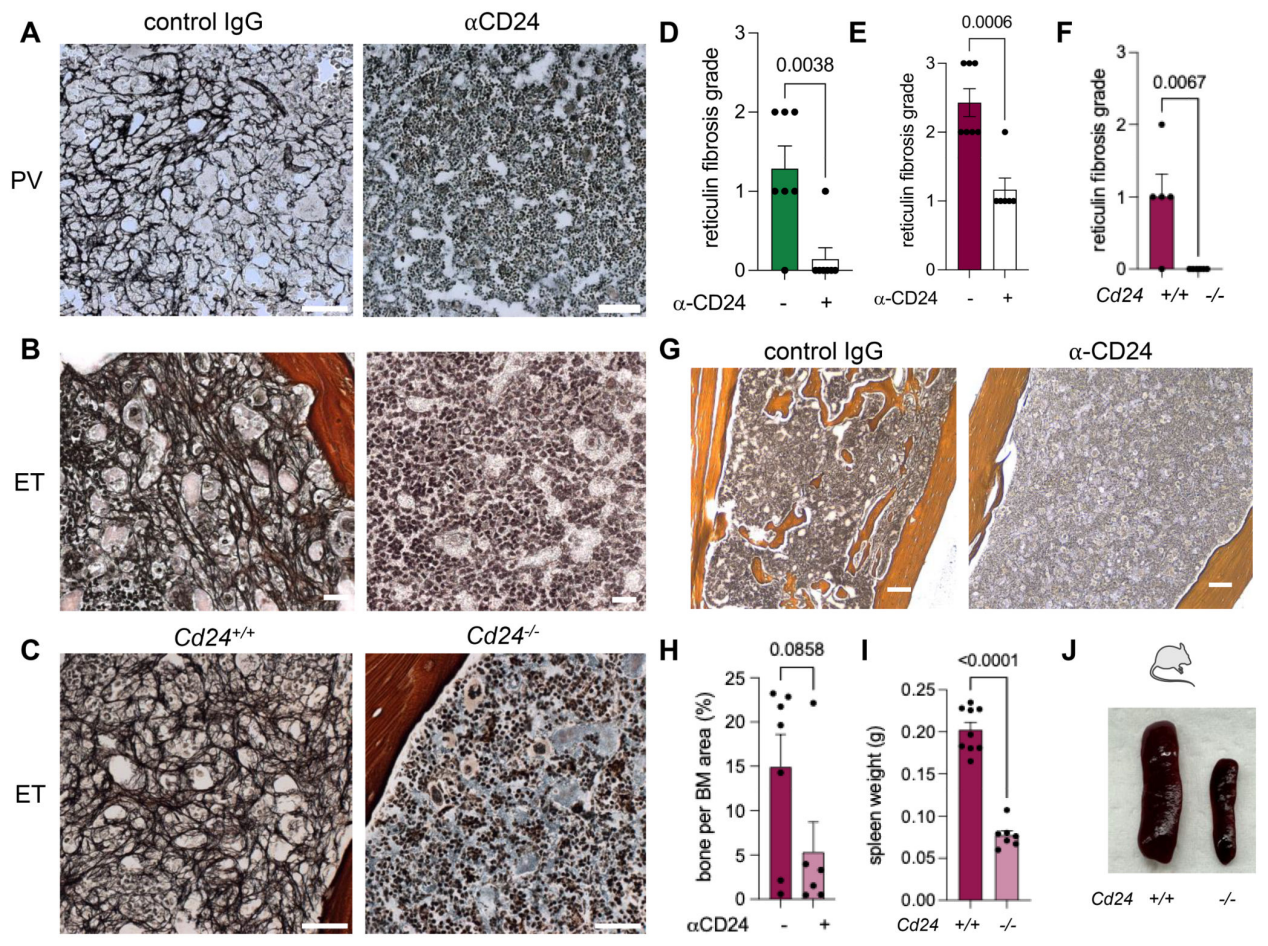
CD24 inhibition prevents myelofibrosis in MPN models. (A-B) BM Gömöri staining of reticulin fibers of MPN models of polycythemia vera (A, PV) or essential thrombocythemia (B, ET) treated for 11w with anti-CD24 blocking antibody (clone M1/69) or isotype control (rat IgG2b) (n=5-7). Scale bar, 40μm. (C) BM Gömöri staining of reticulin fibers 18w after transplantation of WT mice with BM cells from compound *Vav-Cre;JAK2^V617F^ Cd24^-/-^* mice, or control *Vav-Cre;JAK2^V617F^* mice (n=5-7). Scale bar, 40μm. (D-F) BM fibrosis grade in mice in (A-C). (G-H) Osteosclerosis (G) and quantification (H) of bone per BM area in ET-like mice treated for 11w with anti-CD24 blocking antibody (clone M1/69) or isotype control (rat IgG2b) (n=6-7). Scale bar, 100μm. (I-J) Spleen weight (I) and representative pictures (J) in hematopoietic chimeras with/without CD24 deletion 18w after transplantation of WT mice with BM cells from compound *Vav-Cre;JAK2^V617F^ Cd24^-/-^* mice, or control *Vav-Cre;JAK2^V617F^* mice (n=7-9). (D-F, H-I) Data are means±SEM. Each dot is a mouse. Student’s two-tailed unpaired *t* test.

Active TGF-β concentration was higher in media collected from human megakaryocytes co-cultured with *JAK2^V617F^* neutrophils, compared with healthy donors (Figure 6I; supplemental Figure 8D). Futhermore, active TGF-β concentration was similarly elevated in the BM supernatant from ET-like mice (supplemental Figure 8G). However, CD24 blockade in human MPN neutrophils and chronic CD24 blockade or genetic deletion in MPN mice halved active TGF-β (Figure 6J-M), preventing myelofibrosis development in these mice (Figure 7A-F; supplemental Figure 8H).

Excessive bone formation in the BM (osteosclerosis) is frequently observed in myelofibrosis. CD24 blockade reduced osteosclerosis by 3-fold in MPN mice (Figure 7G-H). Moreover, myelofibrosis compromise normal BM hematopoiesis and is associated with extramedullary hematopoiesis in organs such as the spleen, which become abnormally enlarged^6^. Consistent with absent myelofibrosis in MPN mice lacking CD24 (see Figure 7C, F), splenomegaly was not observed in these mice (Figure 7I-J).

Taken together, these findings reveal defective clearance of neutrophils as a cause of pathogenic microenvironmental interactions of inflammatory neutrophils with megakaryocytes, which may increase thrombocytosis and myelofibrosis risk in MPN. Additionally, these results postulate CD24 as a candidate target for innate immune checkpoint blockade in MPN.

## Discussion

Oncogene-independent vulnerabilities are emerging as potential therapeutic targets in cancer. The interaction between cancer cells and their microenvironment shapes disease progression and chemotherapy response in the myeloid malignancies^57^. In MPN, mutant HSC interaction with different BM niches impacts disease development and therapy outcomes^25^, posing the HSC niche as a candidate therapeutic target^35,58^. However, the role of neutrophils in the microenvironmental remodeling in MPN has remained elusive.

Cumulative evidence suggests that neutrophils are markedly heterogeneous and display functions beyond innate immune response, including pro-tumorigenic effects^1,2,59^. However, their interactions with the tumor microenvironment and contributions to cancer progression are incompletely understood. As neutrophils are continuously produced and have short life, their homeostatic clearance is essential to prevent serious consequences, such as chronic inflammation or cancer growth promotion^3^. The prevalent views have considered the overproduction of myeloid cells as the main (or solely) cause of inflammatory cytokines driving microenvironment remodeling in MPN. However, here we found that neutrophils accumulate in MPN not only because they are overproduced, but also due to impaired efferocytosis.

Neutrophil efferocytosis decreases during aging^10^, which is associated with risk of myeloid malignancies, such as MPNs. We found that hyperactive JAK-STAT signaling in MPN neutrophils increases the expression of a ″don’t-eat-me″ signal known for triggering immune evasion in solid tumors^31^. Our results suggest that this function spans to innate immune cells in myeloid malignancies, and particularly neutrophils in MPN. Increased CD24 expression in MPN neutrophils compromises their normal homeostatic efferocytosis and drives their pathogenic interactions with megakaryocytes, likely through binding to P-Selectin. These abnormal interactions promote thrombocytosis and myelofibrosis in MPN, and can be prevented by blocking CD24. These results highlight CD24 as an innate immune checkpoint that can be therapeutically targeted to restore normal clearance of neutrophils and avoid pathogenic cell-cell interactions associated with high risk in MPN.

Immune checkpoint blockade has transformed therapeutic strategies in some hematological malignancies, but has been difficult to achieve in others^60,61^, including MPN^62^. Anti-CD47 strategies have shown promising results in several cancers, but have also faced important limitations, such as anemia caused by widespread CD47 expression, spanning to erythroid cells^63,64^. In contrast, blocking CD24, which is not expressed in human erythroid cells^31^, is not expected to cause anemia in patients. Another promising target proposed is the immunosuppressive PD-L1, which is upregulated in *JAK2^V617F^* cells and can be targeted with nivolumab for T-cell-dependent elimination of mutant cells^65^; however, clinical trials with PD-1 inhibitors nivolumab or durvalumab were terminated due to lack of efficacy or enrolment, respectively^66^. The anti-PD1 antibody pembrolizumab did not induce clinical response in a Phase-II study in MPN^67^, urging the need for alternative immune checkpoint inhibitors. The immunosuppressive cytokine TGFβ has recently emerged as a candidate target in *Calreticulin*-mutated MPN^68^.

Since its discovered function as a ″don’t-eat-me″ signal in solid tumors^31^, CD24 has prompted significant interest as a candidate immunotherapy target in cancer and has been considered as a part of the innate immune checkpoint landscape that influence innate immune activation and response. Recent development of clinical-grade antibodies and antibody conjugates has prompted clinical studies underway, mostly targeting solid tumors. To date, few trials have been completed. In hematology, CD24 blockade has been tested in lymphoid malignancies. In line with our results, CD24 blockade was associated with decreased neutrophil counts, but this effect was not investigated in the context of lymphoproliferative disorders^69^. Our results suggest the potential for repurposing CD24 as a target in myeloid malignancies, and particularly in MPN.

Neutrophils are produced in the BM and released into circulation to patrol peripheral tissues and carry out essential immune functions. Neutrophils are short-lived and, upon aging in circulation, many home back daily to the BM to be cleared by macrophages^7–9^. However, we found that this process fails in MPN due to hyperactive JAK-STAT signaling, which upregulates CD24 in senescent neutrophils, preventing their efferocytosis by macrophages. This does not affect unmutated neutrophils, as functionally-relevant CD24 expression levels require hyperactive JAK-STAT signaling in neutrophils. For this reason, senescent neutrophils do not upregulate CD24 or accumulate in MPN driven by *Calreticulin* gene mutations, which do not trigger hyperactive JAK-STAT signaling in neutrophils.

CD24^hi^ neutrophils accumulating in the BM are inflammatory and affect megakaryocyte function. CD24 can function as a ligand for P-selectin^49^; therefore, it is possible that high CD24 expression in senescent neutrophils favors their interaction with megakaryocytes via P-Selectin. Particularly, high CD24 expression enables *JAK2^V617F^* senescent neutrophils to invade the demarcation membrane system of megakaryocytes through emperipolesis^70^ and increases active TGF-β, which is a key driver of myelofibrosis and osteosclerosis^53,55^.

It is important to note that the role of TGF-β is cell- and -context-dependent, with various (or even opposite) effects on differentiation depending on the stage of commitment, and the local microenvironment and inflammatory milieu^71,72^. During megakaryopoiesis, TGF-β1 regulates myeloid cell proliferation and maturation. Specifically, autocrine TGF-β1 signalling triggers proplatelet formation in megakaryocytes through AKT- and SMAD-dependent pathways^54^. TPO-induced TGF-β1 promotes the buildup of a matrix that sustains thrombopoiesis^73^, supporting an autocrine role for TGF-β1. However, circulating TGF-β1 regulates thrombopoietin secretion from the liver and TGF-β1 depletion in megakaryocytes can instead promote megakaryopoiesis through increased liver-derived thrombopoietin^74^. However, it is difficult to extrapolate these findings to the context of pathological myeloproliferation in MPN.

Our data highlight a potential (previously insufficiently recognized) role of neutrophils in thrombocytosis, through their interactions with megakaryocytes in MPN. Proplatelet formation and platelet release increased when human megakaryocytes were co-cultured with *JAK2^V617F^* neutrophils and circulating platelet counts increased in mice transplanted with *JAK2^V617F^* neutrophils. These observations are consistent with recent findings of neutrophil “plucking” on megakaryocytes, which stimulates platelet production^48^.

The extensive BM microenvironment remodelling in MPN is associated with compromised hematopoiesis, extramedullary hematopoiesis and splenomegaly. Notably, CD24 blockade can restore the normal clearance of senescent neutrophils and intercept with this pathogenic process in experimental models. Interestingly, megakaryocyte emperipolesis of degranulating neutrophils and BM fibrosis are features shared between MPN and grey platelet syndrome^22,75^, suggesting potentially shared mechanisms of BM fibrosis in premalignant and non-malignant hematological diseases, paving the path for future studies. In these disorders, intercepting the emperipolesis of neutrophils might prevent functional alterations in the megakaryocyte causing BM fibrosis.

In summary, this study reveals defective neutrophil clearance as a cause of pathogenic cell-cell interactions that may increase thrombocytosis and myelofibrosis risk, and postulate CD24 as a candidate innate immune checkpoint in MPN.

## Supplementary Material

Supplementary Information is available for this paper.

Visual Abstract

SI Figures

## Data Availability

the original data in this manuscript is available upon reasonable request to the corresponding author.

## Key points

- *JAK2^V617F^* neutrophils evade homeostatic clearance and accumulate in MPN due to JAK2-STAT5-dependent upregulation of CD24.
- CD24 antibody blockade or genetic loss in MPN model restores clearance of neutrophils, improves thrombocytosis and prevents myelofibrosis.
