# Supplementary figures and images for: Defective neutrophil clearance in JAK2V617F myeloproliferative neoplasms drives myelofibrosis via immune checkpoint CD24
Source: Blood. Author manuscript; Available in PMC 2025 Dec 23. (PMC7618514; doi:10.1182/blood.2024027455)

## Slide 1
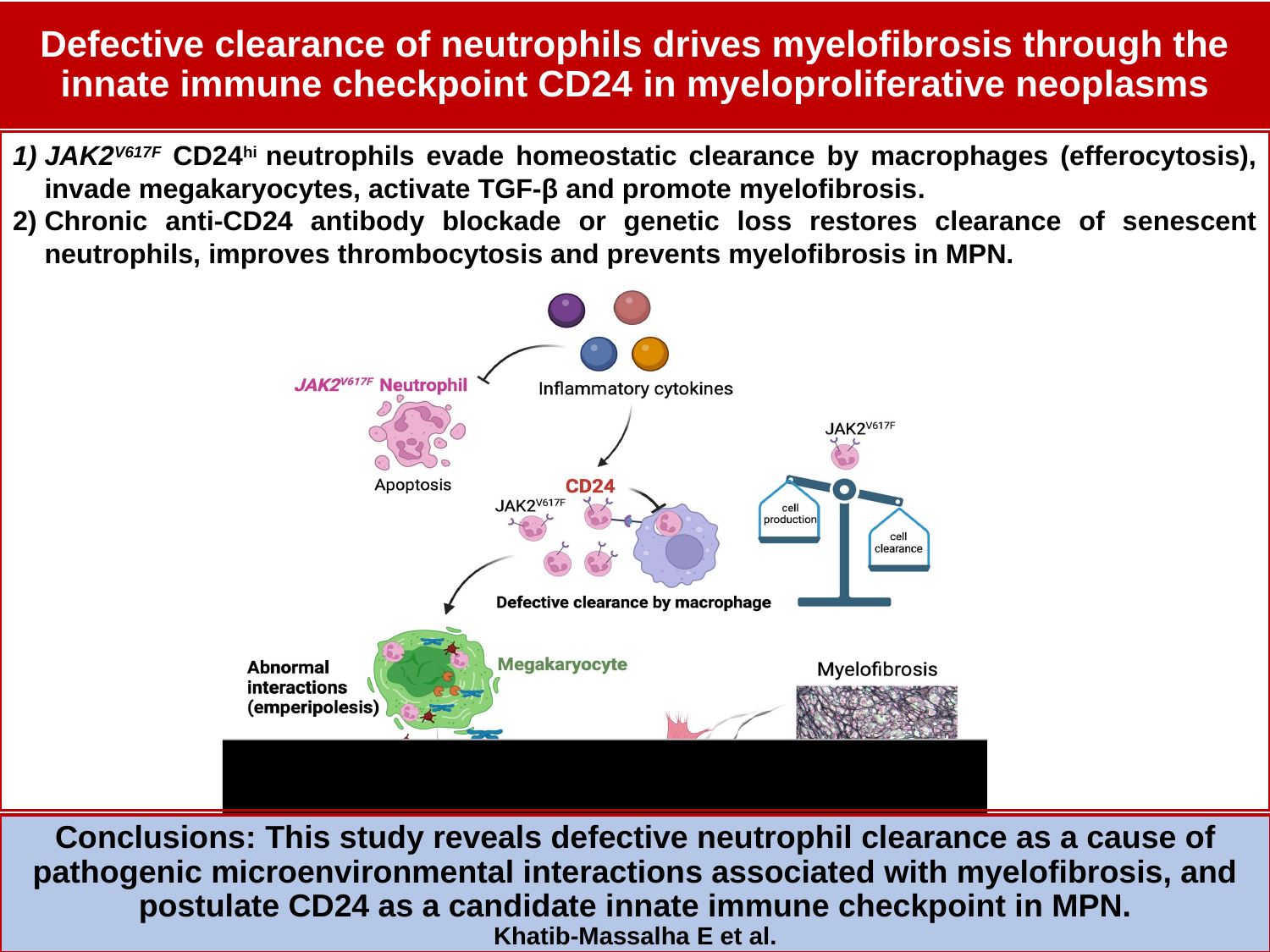

Supplement: Visual Abstract [file EMS211767-supplement-Visual_Abstract.pptx]
