# Supplementary material for: Defective neutrophil clearance in JAK2V617F myeloproliferative neoplasms drives myelofibrosis via immune checkpoint CD24
Source: Blood. Author manuscript; Available in PMC 2025 Dec 23. (PMC7618514; doi:10.1182/blood.2024027455)
Supplement: SI Figures [file EMS211767-supplement-SI_Figures.docx]

**Supplemental Materials and methods**

**Methods**

**Human studies**

All centers had appropriate research and ethical approval. Human peripheral blood samples were obtained from healthy subjects and patients with MPNs after informed consent. Samples were derived from the Cambridge Stem Cell Biobank (REC ID 24/EE/0116, UK), the Andalusian Public Health System Biobank at Virgen del Rocío Hospital (ATM S2400137, Spain) and I.R.C.C.S Policlinico San Matteo Foundation of Pavia (Italy). All research involving human material was approved by Ethical Committees and conducted adhering to the Declaration of Helsinki.

Both male and female patients or healthy controls were included in the different clinical studies. The healthy controls are aged matched. Differences regarding sex were not investigated in this study. The information on the disaggregation between sex and gender was not collected.

**Mouse strains**

Mouse experiments followed protocols approved by the Animal Welfare Ethical Committee, according to the United Kingdom Home Office regulations (PPL P0242B783 and PP1036410). Mice were housed in specific pathogen-free facilities in individually ventilated cages under 12h light–dark cycles and controlled temperature (19–23 °C) and humidity (55 ± 10%) with free access to standard rodent chow (SafeDiet R105-25). The humane endpoint was defined by the project license in accordance with the Home Office regulations as a 15% loss of maximal body weight; all mice were euthanized before or on reaching this stage. *B6.FVB-Tg(Acta2-DsRed)1Rkl/J* (stock no. 31159, The Jackson Laboratory), *LysM-Cre (B6.129P2-Lyz2^tm1(cre)Ifo^/J)* (stock no. 4781, The Jackson Laboratory), *Vwf-TdTomato*^1^, *Vwf-*eGFP^1^, *Vav-Cre;JAK2*^V617F 2^, *Mx1-Cre;JAK2*^V617F^ ^2^, *Mx1-Cre;CALR^del52 3^*, *Stat5*-floxed mice^4^, *C57BL/6-Cd24atm1Pjln/Mmmh* (stock no. 41412-MU, MMRRC) and congenic *B6.SJL-Ptprc^a^Pepc^b^*/BoyJ (CD45.1), CD45.2 C57BL/6 mice (Charles River Laboratories) were used in this study.  In double transgenic *Mx1-Cre;JAK2*^V617F^ mice, Cre activation was induced through intraperitoneal injection of 300 μg/mouse polyinosine-polycytosine (pIpC; catalog no. P1530, Sigma-Aldrich) as a single dose at age 8-9 weeks old, and analyzed 6-8 weeks after induction (for advanced myelofibrosis, mice were analyzed 15-17 weeks after induction). In *Mx1-Cre;Stat5^fl/fl^* mice, Cre activation was induced in experimental and control mice through 6 intraperitoneal injection of pIpC (16mg/kg) and mice were culled after 6x pIpC injection. Mice were sacrificed and cells were harvested in the same timeframe in the morning (8-10AM) to account for the results of the clearance, which occurs preferentially at night.

Both female and male mice were used in different experiments; possible differences regarding sex were not investigated in this study. Mice with similar blood counts were randomly allocated to control or treatment groups.

**Method details**

**BM transplantation**

Age-matched, CD45.2 C57BL/6J mice (8–12 weeks old) were used as recipients in the BM transplantation assays. Recipients were subjected to lethal irradiation (12 Gy whole-body irradiation, split dose 6.0 + 6.0 Gy, 3h apart) before injection. For competitive transplantation, 0.2x10^6^ BM cells from a CD45.2 donor *Mx1-Cre;JAK2*^V617F^ (8 weeks after pIpC injection) and 1.8x10^6^ BM cells from CD45.1 donor were injected into the tail vein of wild-type mice. For non-competitive transplantation, 2 × 10^6^ BM cells from *Vav-Cre;JAK2*^V617F^, *Vav-Cre;JAK2*^V617F^/*Cd24^-/-^* or *Mx1-Cre*;*CALR^del52+^* were injected into wild-type mice. Blood counts (WBC, RBC and platelets) and spleen weight were analyzed from donors before transplantation to determine the disease stage.

***In vivo* treatments**

**Anti-CD24 blocking antibody treatment of ET or PV mice**

First, we confirmed the disease stage before treating the transplanted mice based on their higher levels of blood platelets (ET-like) or RBC (PV-like) 9w post transplantation. PV mice were treated intraperitoneally two times a week for 11 weeks, and ET mice were treated two times for 7 or 11 weeks, with an anti-mouse CD24 mAb (clone M1/69, catalog no. BE0360, BioXCell) or rat IgG2b Isotype control (catalog no. BE0090, BioXCell) at a dose of 200 μg/mouse. Mice were sacrificed 1 day after the last antibody injection.

**Ruxolitinib treatment of ET mice**

Ruxolitinib (Jakavi, Novartis) was resuspended in polyethylene glycol (catalog no. 202371-500g, Sigma-Aldrich) for administration to mice. 8-9 weeks post-transplantation, mice were treated with ruxolitinib (70 mg/kg) or vehicle once daily, two times weekly for 2.5 weeks, as described previously^5^.

**Murine neutrophil isolation**

BM cells were harvested from WT, ET-like or PV-like mice (13-17 week-old ET-like or PV-like mice developed early stage of disease; 18-27 week-old mice developed advanced stage with secondary myelofibrosis). Femora and tibiae were isolated and cleaned of muscles. Bones were then crushed with a mortar and pestle and filtered through a 40µm cell strainer. RBCs were removed using RBC Lysis Buffer (catalog no. 420301, BioLegend). BM cells were resuspended in 200 μl MACS buffer (2 mM EDTA, 0.5% bovine serum albumin in PBS) and neutrophils were isolated following the manufacturer’s protocol (catalog no.130-097-658, Miltenyi). The purity was >94% as determined by flow cytometry. To induce neutrophil ageing ex vivo, isolated neutrophils were incubated at 37°C for 16-18 hours, as previously described^6^.

**Mononuclear cells and granulocyte isolation from human peripheral blood**

Mononuclear cells and granulocyte isolation from human peripheral blood from each healthy control or MPN patient, 18 mL of peripheral venous blood was collected using a 21-G multi-fly needle to avoid lysing cells into S-monovette EDTA vacutainers (S-monovette 9ml K3E REF: 02.1066.001, 1.6mg EDTA/ml). Peripheral blood was then layered slowly onto 20ml Lymphoprep and centrifuged at 1800 rpm for 30 min with brake off. The plasma layer was removed (to within 0.5cm above the MNC layer) and the MNC layer was carefully taken up using a plastic pastette and transferred into a fresh falcon tube, at this point the MNCs were placed into a known volume with PBS/0.5%BSA to enable cell counts and viability to be recorded. The MNCs were then frozen until use for macrophage differentiation. To isolate granulocytes, the remainder of lymphoprep was removed (to within 0.5-1.0 cm of the red cell layer; containing granulocytes). Cold red blood cell (RBC) lysis solution was added to the red cell layer for 5 minutes and then centrifuged at 1,800rpm for 5 mins (RBC lysis step was repeated twice with the second step exposing the red cell pellet to 3 minutes of RBC lysis). The pellet was resuspended in a known volume of PBS/0.5%BSA to enable cell counts and viability to be recorded.  Granulocytes were isolated from individuals diagnosed with ET or PV and carrying the *JAK2^V617F^* mutation (The human JAK2 allele burden in granulocytes was detected as described previously^7^. In Figure 4R-T, neutrophils were isolated from individuals diagnosed with primary myelofibrosis, characterized by the *JAK2^V617F^* mutation with a high allele burden.

**Adoptive transfer of BM neutrophils**

WT mice aged 8-12 weeks were divided into two-three recipient groups. Isolated DsRed^+^ BM WT or *JAK2*^V617F^ neutrophils were aged *ex vivo*. Senescent neutrophils were re-suspended in PBS, and same number (5 × 10^6^) of cells were adoptively transfused each time to recipient mice by tail intravenous injection. For short adoptive transfer, recipient mice were transfused one time with either WT or *JAK2*^V617F^ senescent neutrophils, injected intraperitoneally with IgG or anti-CD24 antibody (200µg per mouse) and sacrificed 24h later. Bones were collected for flow cytometry and histology analysis. For chronic adoptive transfer, recipient mice were transfused (intravenously) with either WT or *JAK2*^V617F^ senescent neutrophils on day 0, 7, 14 and 21, and were bled on day 5, 12, 19 and 23, to monitor circulating platelet counts.

***In vitro* stimulation**

BM isolated neutrophils (1 × 10^6^) from WT, MPN, *Mx1-Cre*;*Stat5^fl/fl^* or their control (treated with pIpC) mice were seeded on 12-well plate in RPMI-1640 culture media (supplemented with 10%FBS and 1% penicillin-streptomycin (P/S)) and then stimulated with either PBS, GM-CSF (100ng/ml), G-CSF (100ng/ml), M-CSF (100ng/ml), IL-1β (100ng/ml), Ruxolitinib (0.5 µM), STAT5 inhibitor (AC-4-130, 5 µM) or STAT3 inhibitor (BP-1-102, 5 μM). Cultured neutrophils were harvested 16-18h post treatment, then washed, stained and analyzed by flow cytometry.

**BM cell extraction, flow cytometry and cell sorting**

Hematopoietic cell isolation from BM or PB was performed as described previously^8^. The marrow was flushed and the bones crushed with a mortar and pestle (catalog no. 10656405, Fisher), in PBS and filtered through a 40-µm strainer (catalog no. 542040, Greiner Bio-One). PB or BM cell suspension was depleted of red blood cells using the RBC lysis buffer for 8 min at 4 °C (catalog no. 420301, BioLegend), washed and numerated using trypan blue (catalog no. 10593524, Fisher). Cells were incubated with the appropriate dilution (2–5 mg ml^−1^) of fluorescent antibody conjugates. DAPI (catalog no. D9542, Sigma-Aldrich) was added to discriminate dead cells. Samples were analyzed with an LSRFortessa flow cytometer (BD Biosciences) or sorted (FACSAria, BD Biosciences) equipped with the FACSDiva Software (BD Biosciences). The following antibodies were used for the staining of the mouse hematopoietic cells in different panels: anti-mouse CD11b (clone M1/70, catalog no. 101216, 101236, 553309, 101211 BioLegend); anti-mouse Ly6G (clone, 1A8, catalog no. 127624, BioLegend); anti-mouse CD62L (clone MEL-14, catalog no. 553152, BD Pharmigen); anti-CXCR4 (clone L276F12, catalog no. 146506); anti-mouse CD24 (clone 30-Fl, catalog no. 138503 or clone M1/69, catalog no. 101821, BioLegend); anti-mouse CD47 (clone miap301, catalog no. 127503, BioLegend); anti-mouse F4/80 (clone BM8, catalog no. 11-4801-82, Invitrogen, eBioscience or catalog no. 123110, BioLegend ); anti-mouse CD41 (clone MWReg30, catalog no. 133904, Biolegend); anti-mouse NK1.1 (catalog no. 108723, BioLegend); anti-mouse CD45.2 (clone 104, catalog no. 109847, BioLegend or 25-0454-U100, TONBO biosciences); anti-mouse CD45.1 (clone A20, catalog no. 60-0453-U100, TONBO biosciences); streptavidin (catalog no. 405241, BioLegend). The following antibodies (BioLegend) used for the staining of mouse hematopoietic stem cells: anti-mouse Sca-1 (catalog no. 108127), anti-mouse c-kit/CD117 (catalog no.105826), anti-mouse CD48 (103422), anti-mouse CD150 (catalog no.115910) and biotin-conjugated lineage cocktail (catalog no.133307, BD Biosciences). The following antibodies (BioLegend) were used for the staining of human neutrophils and platelets in healthy donors or MPN patients: anti-human CD11b (catalog no. 301343), anti-human CD16 (catalog no. 302017); anti-human CD62L (catalog no. 304809), anti-human CD24 (clone ML5, catalog no. 311105); anti-human CD47 (catalog no. 323106); anti-human CD45 (catalog no. 368531). For flow cytometry staining, we used the fluorescence minus one (FMO) as a negative control, which contains all the fluorochromes except for the one that is being measured. All flow cytometry data were analyzed using Kaluza software. The expression levels of proteins (e,g CD24 or CD47) are quantified and presented as “geometric mean”, which is the mean of the fluorescence intensity. Representative flow cytometry gating strategy, plots or histograms were created by Kaluza or Flowjo software. In Figure 3H, CD24 expression presented as fold change from two independent *in vivo* experiments. Different clones of anti-CD24 antibodies were used (M1/69 or 30-Fl). In each experiment, the expression was normalized to the control group.

**CD24 expression in human immature cells**

PBMCs from healthy controls or MPN patients have been defrosted and incubated in an appropriate volume of CD34 Microbead kit Ultrapure human (Miltenyi Biotec, catalog no.130-100-453) according to the manufacturer’s instructions. CD34^+^ cells have been purified using an AutomacsPro (Miltenyi Biotech) and stained with the following antibodies: FITC lineage cocktail (anti-human CD11b (catalog no.101206, clone M1/70, Biolegend), anti-human CD41 (catalog no.303703, clone HIP8, Biolegend), anti-human CD3 (catalog no.561802, clone HIT3a, BD Biosciences), anti-human CD19 (catalog no.392507, clone 4G7, Biolegend), anti-human CD235a (catalog no.349103, clone HI264, Biolegend)), APC anti-human CD34 (catalog no. 343608, clone 561, Biolegend), BV421 anti-human CD38 (catalog no. 303526, Biolegend), PE anti-human CD24 (catalog no. 311106, clone ML5, Biolegend) and Propidium Iodide (catalog no. P1304MP, Invitrogen). Samples were then analyzed with an LSRFortessa flow cytometer (BD Biosciences).

**Apoptosis analysis**

*Annexin V/DAPI staining*. Isolated BM neutrophils from WT or MPN mice were cultured in RPMI media (1 × 10^6^ cells/ml) and treated for 3h with either 10 μg/ml rat anti-mouse CD24 mAb (clone M1/69) or 10 μg/ml isotype antibody (rat IgG2b) from BioXcell. After 3h, cells were collected for staining. For cross-linking CD24, isolated BM neutrophils from MPN mice were cultured and treated for 22h with primary anti-CD24 antibody, control IgG or with crosslinked primary-secondary antibodies. Primary and goat anti-rat secondary (catalog no. 112-005-003; Jackson ImmunoResearch) antibodies pre-incubated before addition to cells at a 2:1 ratio to ensure efficient cross-linking of primary antibody and to ensure no excess secondary antibody present in the culture. Isolated neutrophils were stained first with cell surface markers (CD11b, Ly6G), and then resuspended in Annexin V binding buffer. Apoptosis was measured using FITC AnnexinV (catalog no. 640945, BioLegend) and DAPI, and cells were analyzed by flow cytometry.

**Mouse *in vitro* phagocytosis assay**

**Macrophage generation and stimulation**

BM cells were harvested from 10- to 15-week-old WT mice. Femurs and tibiae were isolated and cleaned of muscles. Bones were then crushed with a mortar and pestle, and a uniform cell suspension was made by extensive passage of marrow through a 40µm cell strainer. RBCs were removed using RBC Lysis Buffer (catalog no. 420301, BioLegend). BM macrophages were generated by culturing the BM cells in DMEM/F-12, HEPES (catalog no. 11330032, Gibco) with 10% FBS, 1%P/S and 25 ng/mL murine M-CSF (catalog no. 315-02, Peprotech). On day 4, cells were detached and replated in a 12-well plate with 10^5^ cells per well with 1 ml medium and M-CSF and were kept until use on day 7.

**Neutrophil preparation**

One day before the phagocytosis assay, neutrophils were isolated from WT or MPN mice (as described above in “Neutrophil isolation” section) and cultured in RPMI-1640 (+10%FBS+ 1%P/S) overnight prior to co-culture, to induce their aging. On the day of assay, the number of macrophages per well was counted with a hemocytometer. Senescent neutrophils (carrying β-actin-DsRed) or fluorescently labelled by incubation with 5µM Cell-Tracker Green CMFDA (catalog no. C7025, Invitrogen) for 20 mins in the dark at 37°C and washed with RPMI serum-free medium. Where indicated, 10µg/ml of anti-mouse CD24 mAb (clone M1/69, catalog no. BE0360, BioXCell), 10µg/ml of anti-mouse CD47 mAb (clone MIAP410, catalog no. BE0283) or 10µg/ml of rat IgG2a isotype control (catalog.no BE0089, BioXcell) was added to macrophage cultures immediately before co-culture. Then, senescent neutrophils were added to each macrophage culture to achieve a 4:1 target-to-effector cell ratio. Phagocytic macrophages were analyzed 3h post co-culture (either by flow cytometry (Figure 1C-D and supplemental Figure 2I) or by live cell imaging on an Incucyte system (Figure 1G, Figure 2A-C, D-F, supplemental Figure 2G-H and Figure 3J-M). Phagocytic (DsRed or green CMFDA positive) macrophages were quantified with at least two randomly selected fields of view for each condition (live imaging).

**Human *in vitro* phagocytosis assay**

Human macrophage generation and stimulation: Peripheral mononuclear cells (PBMCs) were obtained from the buffy coats of healthy donors by the Human Research and Human Tissue Authorities. PBMCs were then pelleted by centrifugation at 300 g for 5 min, resuspended in a RPMI-1640 medium consisting of 20% FBS and 10% DMSO, and cryopreserved until use. Monocytes were isolated from PBMCs through adherence to plastic and then differentiated into macrophages by 7-9 days of culture in IMDM + 10% human AB serum (catalog no. H5667, Sigma). Macrophage differentiation was induced by adding 25 ng/mL of human M-CSF (catalog no. 300-25, Peprotech) on day 1. Half of the medium was replaced on day 4, and from that day onwards, three human cytokines (20ng/mL IL-4, 50ng/mL IL-10, and 50ng/mL TGF-β1) from Peprotech (catalog no. 200-04, 200-10, and 100-21, respectively) were added daily. Additionally, two PBS washes followed by complete medium replacement were performed one day before co-culture.

**Human granulocytes preparation**

Fresh granulocytes from healthy donors or MPN patients were obtained alongside the PBMCs above and immediately cultured upon receipt in RPMI-1640 (+10% FBS + 1% P/S) overnight prior to co-culture, to induce their aging.

**Phagocytosis assay**

On the day of assay, the number of macrophages per well was counted with a hemocytometer. Senescent granulocytes were labelled by incubation with 5µM Cell-Tracker Green CMFDA (catalog no. C7025, Invitrogen) for 20 min in the dark at 37°C and washed with RPMI serum-free medium. Where indicated, 10µg/ml of anti-human CD24 antibody (clone SN3 (catalog no. NB100-64861, Novus) or mouse IgG1 isotype control (catalog.no BE0083, BioXcell) was added to macrophage cultures immediately before co-culture. Then, four times the number of granulocytes were added to each macrophage culture to achieve a 4:1 target-to-effector cell ratio. Phagocytic macrophages were analyzed 3h post co-culture (by live cell imaging on an Incucyte system). Phagocytic (CMFDA positive) macrophages were manually quantified with at least two randomly selected fields of view for each condition ImageJ software.

**Mouse *in vitro* emperipolesis assay**

**Megakaryocyte generation and stimulation**

BM cells were harvested from 10- to 15-week-old mice. Bones were cleaned and flushed, and a uniform cell suspension was made by extensive passage of marrow through a 40µm cell strainer. RBCs were removed using RBC Lysis Buffer (catalog no. 420301, BioLegend). BM cells were incubated with biotin-linage Cocktail for 20 mins on ice and washed with magnetic buffer (PBS+0.5%BSA+2mM EDTA). Cells were incubated with BDIMag StrepParticle Plus and magnetic buffer for 30 mins on ice. Next, magnetic buffer was added to depleted cells on magnet for 5 min in 4°C. Lineage negative cells were cultured in Cellgro medium with 50ng/ml TPO for 4-5 days.

Neutrophil preparation: one day before the emperipolesis assay, neutrophils were isolated from DsRed WT or MPN mice (as described above in “Neutrophil isolation” section) and cultured in RPMI-1640 (+10% FBS+1% P/S) overnight prior to co-culture.

On the day of assay, the number of megakaryocytes per well was counted with a hemocytometer. Senescent neutrophils were added to each megakaryocyte culture to achieve a 4:1 neutrophil-to-megakaryocyte ratio. Cells in co-culture were treated with anti-CD24 blocking antibody (10μg/ml), anti-P-selectin antibody (RB40.34; 20μg/ml) or isotype control (10μg/ml). Emperipolesis of neutrophils were imaged 3h post co-culture by confocal microscopy and were quantified with at least two randomly selected fields of view for each condition by using ImageJ software.

Emperipolesis quantification was defined as described previously^9^. For each area, 30-70 megakaryocytes were counted. Results are expressed as percentage of megakaryocytes with emperipolesis (presence of neutrophils within megakaryocytes was indicated as emperipolesis).

**Human *in vitro* emperipolesis and platelet production assay**

**Human Megakaryocyte differentiation**

Human hematopoietic stem and progenitor cells (HSPCs) were obtained from healthy donors. CD34^+^ HSPCs were selected by an immune-magnetic bead selection kit (CD34 MicroBead Kit, catalog no. 130-042-201, Miltenyi Biotec), and cultured in Stem Span medium (StemCell Technologies) supplemented with 1% penicillin-streptomycin, 1% L-glutamine, 10 ng/ml human IL-11 and 10 ng/ml or 100 ng/mL human TPO (Peprotech), at 37 °C in a 5% CO_2_ fully humidified atmosphere, as previously described^10,11^. The medium change was performed every 3 days.

**Neutrophil isolation**

Human granulocytes were obtained from healthy donors or *JAK2^V617F^* MPN patients (as described above). In all co-culture experiments neutrophils from controls and MPN patients were processed in parallel and cultured with megakaryocytes from the same healthy donor.

**Preparation of the silk fibroin solution**

Silk fibroin aqueous solution was obtained from B. mori silkworm cocoons as previously described^10^. Briefly, fibroin was obtained by boiling cocoons in Na_2_CO_3_. The fibers were rinsed in ultrapure water and dried at room temperature (RT) for 48 h. The dried fibers were subsequently solubilized in Lithium Bromide solution for 4h at 60°C. The silk solution was then dialyzed against deionized water using a Slide-A-Lyzer cassette (Thermo Fisher).

**Silk BM model fabrication, assembly, and perfusion**

To reproduce the spongy architecture and composition of the BM niche, the silk solution (8% w/v) was mixed with 25 μg/ml human fibronectin (Merck Life Science) and dispensed into a molding chamber. NaCl particles (approximately 500 μm in diameter) were then sifted into the solution. The scaffolds were placed at RT for 48h and finally soaked in distilled water to leach out the NaCl particles. The scaffolds were sterilized in 70% filtered ethanol. Scaffolds were rinsed in the culture medium before being used.

The dynamic perfusion of the silk BM scaffold was performed by using a peristaltic pump (ShenChen Flow Rates Peristaltic Pump - LabV1) connected to a bioreactor chamber manufactured using 3D SLA printing technology. The chamber was designed using CAD software (Fusion 360) to generate STL files (Standard Triangulation Language) that were then exported to the SLA 3D printer Form 3B (Formlab) for fabrication. The printing was performed using the Biomed Clear biocompatible resin cured in layers of 100 µm. The resulting bioreactor chamber was washed with isopropyl alcohol (IPA) to remove the unpolymerized resin and then cured in a UV oven for 1 h. The resulting flow chamber consists of four wells of 10 (L) × 5 (W) × 5 (H) mm having independent inlets and outlet channels. Each well was equipped with a silk fibroin scaffold. At day 13 of differentiation, 1×10^5^ megakaryocytes were seeded into each silk scaffold alone or in co-culture with 4×10^5^ neutrophils and kept overnight at 37 °C and 5% CO_2_. Then, samples have been perfused for 4 hours to allow platelet collection into transfusion bags, as previously described. In some experiments, before being seeded, megakaryocytes were labelled with 0.5 μM CFSE (Life Technologies), for 15 min, at room temperature; while neutrophils were labelled with 0.5 μM Deep-Red (Life Technologies), for 15 min, at room temperature. Samples were washed 2 times with culture media before being dispensed into the silk scaffolds. For confocal imaging the chamber was equipped with a glass window at the bottom.

**Flow cytometry**

For the analysis of megakaryocyte differentiation, 5×10^4^ cells at the end of differentiation were suspended in phosphate-buffered saline (PBS) and stained with lineage-specific markers CD42b (catalog no. IM0648U or catalog no. IM1417U) and/or CD41 (catalog no. B16894) (Beckman Coulter), at RT in the dark for 20 min. For the analysis of neutrophil, 5×10^4^ cells were stained with CD45 (catalog no. 368531), CD11b (catalog no. 301344), CD16 (catalog no. 302018), CD62L (catalog no. 304810) (BioLegend), at 4°C in the dark for 20 min.

Megakaryocyte/platelet-neutrophil interaction was assessed after separate labelling with CD42b and CD62L/CD66b, respectively. Analysis was performed at time 0, and after 1, 2, 3 hours of incubation into culture medium, at 37 °C and 5% CO_2_.

Flow cytometry settings for *ex vivo* generated platelets were established, as previously described^12^. Briefly, collected platelets produced were analyzed using the same forward and side scatter pattern as human peripheral blood and identified as CD61^+^ (catalog no. IM1758, Beckman Coulter) events. Isotype controls were used as a negative control to exclude non-specific background signals. The platelet number was calculated using a Tru-Count bead standard (Becton Dickinson).

All samples were acquired using a FACSLyric flow cytometer (Becton Dickinson). A minimum of 5,000 events was acquired. Off-line data analysis was performed using the Kaluza software package (Beckman Coulter).

**Imaging of the 3D silk BM model**

For immunofluorescence imaging of megakaryocyte-neutrophil emperipolesis and proplatelet formation within the silk BM scaffold, samples were fixed in 4% PFA for 20 min and then blocked with 5% bovine serum albumin (BSA, Sigma) for 30 min at RT. Samples were permeabilized with 1% Triton X-100 for 10 minutes, and then stained with anti-CD61 (catalog no. IM0540, Beckman Coulter, 1:100), anti-CD66b (catalog no. bs-0744R, Bioss Antibodies,1:50), anti-elastase (catalog no. PA5-115648, Invitrogen, 1:200) and/or anti-myeloperoxidase (catalog no. MA5-32582, Invitrogen, 1:100), diluted in 1% BSA, overnight at 4 °C. Samples were finally immersed in Alexa Fluor secondary antibody (1:200) diluted in 1% BSA for 2h at RT. Nuclei were stained with Hoechst. Silk fibroin fluorescence was brightened by Hoechst. 3D reconstruction and image processing were performed using Leica and Arivis software.

The quantification of mean fluorescence intensity (MFI) in Figure 6C-H has been performed by FIJI ImageJ software. CD61-positive megakaryocytes were identified to delineate the specific region of interest (ROI). Subsequently, the total fluorescence intensity for CD61, elastase, and/or myeloperoxidase was measured within the selected region and expressed as the MFI ratio per single cell (MFI ratio has been calculated for elastase/CD61 and myeloperoxidase/CD61). To address nonspecific fluorescence background, negative controls were routinely performed by omitting both secondary and primary antibodies from different sections of the same 3D sample.

***In vivo* macrophage depletion**

12-15 week-old *Vwf-tdtomato* MPN or control mice were injected into the tail vein with PBS-liposome control or clodronate liposomes (250µl/mouse, catalog no. CP-005-005, LOPOSOMA research) and sacrificed 24h later. Femurs were collected for histology.

**Histology of mouse bones**

Femurs and tibias were collected, cleaned and put in PBS, 2% paraformaldehyde (PFA) (Sigma-Aldrich) overnight. For cryosectioning and immunostaining, bones were washed once with PBS, decalcified in 250 mM EDTA and PBS for 14 days at 4°C, put in 15% sucrose and PBS for 24h then in 30% sucrose and PBS for another 24h, and embedded with OCT (catalog no. 12678646, Thermo Fisher Scientific) in plastic cryomolds (catalog no. 4557, Sakura). The samples were stored at −80 °C and 12µm-thick sections were obtained using a cryostat (Leica Biosystems). Alternatively, after fixation bones were embedded in paraffin and 3µm-thick sections were stained with reticulin for conventional fibrosis and osteosclerosis evaluation. Images were acquired with a microscope (Zeiss Apotome) using 10×, 20× and 40× objectives and analyzed with ImageJ. Osteosclerosis quantification was performed using the NDP.view2 software (Hamamatsu).

**Immunofluorescence of cryosections**

Immunofluorescence staining of cryosections was performed as described previously^13^ using femurs, with minor modifications. Briefly, cryosections of 12 µm were incubated with Tris-NaCl-blocking (TNB) buffer (0.1 M Tris-HCl, pH 7.5, 0.15 M NaCl, 0.5% blocking reagent, PerkinElmer) at RT, for 1 hour. Samples were incubated with conjugated antibodies: anti-Ly6G (1:200, catalog no. 127610, BioLegend); anti-CD41 (1:200, catalog no. 133904, BioLegend); anti-CD169 (1:200, catalog no. 142419) diluted in PBS overnight at 4 °C. Samples were rinsed with PBS four to five times for 10 min. Stained tissue sections were counterstained for 10 min with 5 mM DAPI in PBS and rinsed with PBS. For sections, slides were mounted in mounting medium (catalog no. S3023, DAKO). Images were acquired with a confocal microscope (Stellaris, ZEISS 980 or Zeiss Apotome) using 10×, 20× and 40× objectives and analyzed with ImageJ. At least two independent and randomly selected BM areas were imaged and analyzed per sample. Emperipolesis quantification was defined as described previously^9^. For each area, 30-70 megakaryocytes were counted. Results are expressed as percentage of megakaryocytes with emperipolesis (presence of neutrophils within the megakaryocytes was indicated as emperipolesis).

**Active TGF-β and GM-CSF quantification**

Mouse active TGF-β protein amount (catalog no.437707, BioLegend) or GM-CSF (catalog no. MGM00, R&D) were measured in BM samples using commercial ELISA reagents, following the manufacturer’s protocol. Total protein content in central BM fluids was quantified by BCA assay. Active TGF-β or GM-CSF concentration in the BM fluids was normalized per protein content. Human active TGF-β (catalog no.437707, BioLegend) was measured in medium collected from the 3D silk BM model upon overnight co-culture of megakaryocytes with neutrophils from heathy donors or MPN patients.

**Multiplex analysis of mouse bone marrow fluids**

Mouse inflammatory cytokines were measured in BM samples using Milliplex MAP Kit Mouse Cytokine/Chemokine Magnetic Bead Panel (Millipore, Billerica, MA) according to manufacturer׳s specifications. The cytokines chosen for analysis were IL-4, IL-6, IL-10 and IL-17. Cytokines concentration in the BM fluids was normalized per protein content.

**Data processing**

The oligonucleotide microarrays data of neutrophils from MPN patients^14^ was downloaded from Gene Expression Omnibus with reference series number GSE54646. The differential expression analysis was performed using limma^15^. Genes with P value less than 0.1 are considered as differentiated expressed genes. The whole-transcriptome sequence data of neutrophil from MPN patients^16^ was downloaded from the European Genome-phenome Archive with the accession number EGAS00001003486. Raw reads were aligned by HISAT2 using Genome Reference Consortium Human Build 38^17^, followed by gene read count quantification using FeatureCounts^18^. Count reads were normalized by DeSeq2^19^. To investigate the transcriptomic discrepancy between the neutrophils from CD24-high MPN patients (with top 25% CD24 expression level in the cohort) and CD24-low MPN patients (with bottom 25% CD24 expression level in the cohort), gene set enrichment analyses (GSEA) were performed using a weighted statistic, ranking by signal to noise ratio, 1000 gene-set permutations, and molecular signature databases (Hallmarks, Gene Ontology and Reactome pathway databases)^19^. In parallel, gene set variation analysis (GSVA), a sample-wise gene set enrichment analysis^20^ was used to score the pathway activity of each myelofibrosis patient. The statistical relationship of pathway activity for each patient with the genes of interest was evaluated using Spearman's rank correlation.

**Statistics, reproducibility and analysis**: Data distribution was assumed to be normal, but this was not formally tested. No statistical method was used to predetermine sample size, but our sample sizes are similar to those calculated for similar experiments in previous publications. No animals or data points were excluded from the analyses.

**Data Availability statement**: The data that support the findings of this study are included in the paper or available from the corresponding author upon reasonable request.

**Code Availability statement**: This study did not generate new code.

**Reagents and Resources**

| **REAGENT or RESOURCE** | **SOURCE** | **IDENTIFIER** |
| --- | --- | --- |
| **Antibodies** | | |
| anti-mouse CD24 mAb (clone M1/69) | BioXCell | Cat#BE0360; RRID: AB_2894779 |
| anti-mouse CD47 mAb (clone MIAP410) | BioXCell | Cat#BE0283; RRID: AB_2687806 |
| rat IgG2b Isotype control | BioXCell | Cat# BE0090; RRID:  AB_1107780 |
| anti-mouse P-selectin mAb (clone RB40.34) | BD Biosciences | Cat# 742128, RRID:AB_2871391 |
| PE/Cyanine7 anti-mouse CD11b (clone M1/70) | BioLegend | Cat#101216; RRID: AB_312799 |
| Brilliant Violet 421 anti-mouse CD11b (clone M1/70) | BioLegend | Cat#101236, RRID: AB_11203704 |
| APC anti-mouse CD11b (clone M1/70) | BioLegend | Cat#101211; RRID:  AB_312794 |
| Biotin anti-mouse CD11b (clone M1/70) | BD Biosciences | Cat#553309; RRID: AB_394773 |
| APC/Cyanine7 anti-mouse Ly6G (clone, 1A8) | BioLegend | Cat#127624; RRID: AB_10640819 |
| Alexa Fluor® 647 anti-mouse Ly6G (clone, 1A8) | BioLegend | Cat#127610; RRID: AB_1134159 |
| APC anti-mouse CD62L (clone MEL-14) | BD Pharmigen | Cat#553152; RRID: AB_398533 |
| PE anti-CXCR4 (clone L276F12) | BioLegend | Cat#146506; RRID:  AB_2562783 |
| PE anti-mouse CD24 (clone 30-Fl) | BioLegend | Cat#138503; RRID: AB_10576359 |
| PE/Cyanine7 anti-mouse CD24 (clone M1/69) | BioLegend | Cat#101821; RRID: AB_756047 |
| Alexa Fluor(R) 488 anti-mouse CD169 | BioLegend | Cat#142419; RRID: AB_2566436 |
| FITC anti-mouse CD47 (clone miap301) | BioLegend | Cat#127503; RRID: AB_1134129 |
| FITC anti-mouse F4/80 (clone BM8) | eBioscience | Cat#11-4801-82; RRID: AB_2637191 |
| PE anti-mouse F4/80 (clone BM8) | BioLegend | Cat#123110; RRID: AB_893486 |
| FITC anti-mouse CD41 (clone MWReg30) | Biolegend | Cat#133904; RRID: AB_2129746 |
| APC/Cyanine7 anti-mouse NK1.1 (clone PK136) | Biolegend | Cat#108723; RRID: AB_830870 |
| anti-mouse CD45.2 (clone 104) | BioLegend | Cat#109847; RRID: AB_2616859 |
| anti-mouse CD45.2 (clone 104) | TONBO biosciences | Cat#25-0454-U100; RRID: AB_2573350 |
| APC anti-mouse Siglec-g (clone SH2.1) | eBioscience | Cat#17-5833-80 |
| Biotin anti-mouse lineage cocktail | BD Biosciences | Cat#51-9000794 |
| BV421 anti-mouse Sca-1 | BioLegend | Cat#108127 |
| APC-Cy7 anti-mouse c-kit/CD117 | BioLegend | Cat#105826 |
| APC anti-mouse CD150 | BioLegend | Cat#115910 |
| PerCP/Cy5.5 anti-mouse CD48 | BioLegend | Cat#103422 |
| Brilliant Violet 711 Streptavidin | BioLegend | Cat#405241 |
| FITC-Annexin V | BioLegend | Cat#640945; RRID: AB_2629519 |
| Brilliant Violet 711 anti-human CD11b | BioLegend | Cat#301343; RRID: AB_2563520 |
| Brilliant Violet 711 anti-human CD11b | BioLegend | Cat#301343; RRID:  AB_2563792 |
| FITC anti-human CD11b (clone M1/70) | BioLegend | Cat#101206; RRID:  AB_312789 |
| FITC anti-human CD41 (clone HIP8) | BioLegend | Cat#303703; RRID:  AB_314373 |
| FITC anti-human CD3 (clone HIT3a) | BD Biosciences | Cat#561802; RRID:  AB_10893003 |
| FITC anti-human CD19 (clone 4G7) | BioLegend | Cat#392507; RRID:  AB_2750098 |
| FITC anti-human CD235a (clone HI264) | BioLegend | Cat#349103; RRID:  AB_10612923 |
| APC anti-human CD34 (clone 561) | BioLegend | Cat#343608; RRID:  AB_2228972 |
| BV421 anti-human CD38 | BioLegend | Cat#303526; RRID:  AB_10983072 |
| APC/Cyanine7 anti-human CD16 (clone 3G8) | BioLegend | Cat#302017; RRID: AB_314217 |
| APC/Cyanine7 anti-human CD16 (clone 3G8) | BioLegend | Cat#302018; RRID: AB_314218 |
| APC anti-human CD62L (clone DREG-56) | BioLegend | Cat#304809; RRID: AB_314469 |
| APC anti-human CD62L (clone DREG-56) | BioLegend | Cat#304810; RRID: AB_314470 |
| PE anti-human CD24 (clone ML5) | BioLegend | Cat#311105; RRID: AB_314854 |
| FITC anti-human CD47 (clone CC2C6) | BioLegend | Cat#323106; RRID: AB_756136 |
| PE/Cyanine7 anti-human CD45 (clone 2D1) | BioLegend | Cat#368531; RRID: AB_2715891 |
| Anti-human CD24 (clone SN3) | Novus | Cat#NB100-64861 |
| mouse IgG1 isotype control | BioXcell | Cat#BE0083; RRID: AB_1107784 |
| FITC- anti-human CD42b | Beckman Coulter | Cat#IM0648U |
| PE- anti-human CD42b | Beckman Coulter | Cat#IM1417U |
| APC-anti-human CD41 | Beckman Coulter | Cat#B16894 |
| Anti-human CD61 | Beckman Coulter | Cat#IM0540 |
| Anti-human CD66b | Bioss Antibodies | Cat#bs-0744R |
| Anti-human elastase | Invitrogen | Cat#PA5-115648; RRID: AB_2900283 |
| Anti-human myeloperoxidase | Invitrogen | Cat#MA5-32582; RRID: AB_2809859 |
| **Biological samples** | | |
| Human healthy or MPN neutrophils | Cambridge Biobank | N/A |
| Human healthy MNCs | Cambridge Biobank | N/A |
| Human healthy or MPN neutrophils | Andalusian Public Health System Biobank at Virgen del Rocío Hospital | N/A |
| Human healthy peripheral blood mononuclear cells | Andalusian Public Health System Biobank at Virgen del Rocío Hospital | N/A |
| Human healthy or MPN neutrophils | Pavia Biobank | N/A |
|  |  |  |
| **Chemicals, peptides, and recombinant proteins** | | |
| ruxolitinib | Jakavi, Novartis | N/A |
| polyethylene glycol | Sigma-Aldrich | Cat#202371-500g |
| clodronate liposomes | LOPOSOMA research | Cat#CP-005-005 |
| Recombinant Murine GM-CSF | Peprotech | Cat# 315-03 |
| Recombinant Murine IL-1β | Peprotech | Cat# 211-11B |
| Recombinant Murine G-CSF | Peprotech | Cat# 250-05 |
| Recombinant Murine M-CSF | Peprotech | Cat# 315-02 |
| Recombinant Murine TPO | Peprotech | Cat# 315-14 |
| Recombinant Human TPO | Peprotech | Cat# 300-18 |
| Recombinant Human GM-CSF | Peprotech | Cat# 300-03 |
| Recombinant Human M-CSF | Peprotech | Cat# 300-25 |
| Recombinant Human IL-11 | Peprotech | Cat# 200-11 |
| Recombinant Human IL-4 | Peprotech | Cat#200-04 |
| Recombinant Human IL-10 | Peprotech | Cat#200-10 |
| Recombinant Human TGF-β1 | Peprotech | Cat#100-21 |
| STAT5 inhibitor; AC-4-130 | MedChemExpress | Cat#AC-4-130 |
| STAT3 inhibitor; BP-1-102 | Selleck Chemicals | Cat#S7769 |
| Fetal Bovine serum (FBS) | N/A | N/A |
| Penicillin-Streptomycin | N/A | N/A |
| RMPI-1640 medium | Sigma-Aldrich | Cat#R8758 |
| DMEM/F12 medium | Gibco | Cat#11330-032 |
| Cellgro medium | CellGenix | Cat#20802-0500 |
| Stem Span medium | StemCell Technologies | Cat#09600 |
| Paraformaldehyde | Sigma-Aldrich | Cat#158127 |
| DAPI | Thermo Fisher Scientific | Cat#D1306 |
| DAKO Fluorescence Mounting Medium | Agilent | Cat#S3023 |
| TNB (0.1 M Tris–HCl, pH7.5, 0.15 M NaCl, 0.5% blocking reagent) | Perkin Elmer | Cat#FP1020 |
| Triton X-100 | Sigma-Aldrich | Cat#T8787 |
| RBC Lysis Buffer | BioLegend | Cat#420301 |
| Bovine Serum Albumin | Sigma-Aldrich | Cat#A7906 |
| Albumin standard | ThermoFisher scientific | Cat#23209 |
| CellTracker™ green CMFDA Dye | Invitrogen | Cat#C7025 |
| Trypan blue | Thermo Fisher scientific | Cat#10593524 |
| OCT embedding cryoembedding Matrix | Thermo Fisher scientific | Cat#12678646 |
| **Critical commercial assays** | | |
| Neutrophils isolation kit | Miltenyi Biotec | Cat#130-097-658 |
| Mouse active TGF-β ELISA kit | BioLegend | Cat#437707 |
| Mouse GM-CSF ELISA kit | R&D | Cat#MGM00 |
| Milliplex MAP Kit Mouse Cytokine/Chemokine Magnetic Bead Panel | Millipore | N/A |
| CD34 MicroBead Kit | Miltenyi Biotec | Cat#130-042-201 |
| CD34 Microbead kit Ultrapure human | Miltenyi Biotec | Cat#130-100-453 |
| **Experimental models: Organisms/strains** | | |
| Mouse: *B6.FVB-Tg(Acta2-DsRed)1Rkl/J* | The Jackson Laboratory | JAX: 031159 |
| Mouse: *Vwf-TdTomato* | Carrelha et al., 2018 | REF^1^ |
| Mouse: *Vwf-eGFP* | Carrelha et al., 2018 | REF^1^ |
| Mouse: *Vav-Cre;JAK2*^V617F^ | Tiedt, R. et al., 2008 | REF^2^ |
| Mouse: *Mx1-Cre;JAK2*^V617F^ | Tiedt, R. et al., 2008 | REF^2^ |
| Mouse: *Mx1-Cre; CALR^del52+^* | Li, J. et al., 2018 | REF^3^ |
| Mouse: *Cd24* KO (*C57BL/6-Cd24atm1Pjln/Mmmh*) | MMRRC | 041412-MU |
| Mouse: *LysM-Cre (B6.129P2-Lyz2^tm1(cre)Ifo^/J)* | The Jackson Laboratory | JAX:004781 |
| Mouse: congenic CD45.1 C57BL/6 mice | Charles River Laboratories | N/A |
| Mouse: CD45.2 C57BL/6 mice | Charles River Laboratories | N/A |
| Mouse: *Stat5*-floxed | Cui, Y. et a., 2004 | REF^4^ |
| **Software and algorithms** | | |
| Image J/Fiji Software | National Institutes of Health | RRID:SCR_002285 |
| GalliosTM Kaluza Software | BeckmanCoulter | RRID:SCR_016700 |
| Flowjo 10.8.1 Software | FLOWJO | RRID:SCR_008520 |
| GraphPad Prism 9 or 10 | GraphPad Software |  |
| NDP.view 2 | Hamamatsu | U12388-01 |
| R software | <https://www.r-project.org/> |  |
| GSEA software | <https://www.gsea-msigdb.org/gsea/index.jsp> |  |

**Supplementary Figure 1. Senescent neutrophils accumulate in MPN BM due to defective clearance.**

(A) Frequency of BM senescent neutrophils (Ly6G^+^CD62L^lo^ or CXCR4^hi^CD62L^lo^) from MPN mice (n=3).

(B, J, K) Gating strategy of (B) mouse BM neutrophils and senescent neutrophils, (J) phagocytic macrophages, and (K) human blood neutrophils and senescent neutrophils.

(C) BM CXCR4 protein expression in senescent neutrophils from WT (n=6) or MPN (n=3) mice.

(D-F) Analysis of senescent (Ly6G^+^CD62L^lo^) and young (Ly6G^+^CD62L^hi^) neutrophils. Expression of (D) CD11b or (E) CD45 in CD62L^lo^ or CD62L^hi^ MPN neutrophils (n=8-9). (F) Frequency of forward side scatter (FCS) of CD62L^lo^ or CD62L^hi^ MPN neutrophils (n=8-9).

(G-H) Frequency of (G) total or (H) senescent neutrophils in WT mouse circulation and BM niches close to the bone surface (endosteal) or further away from bone (central) (n=3-4 mice).

(I) Experimental workflow for *in vitro* phagocytosis assay of WT or MPN macrophages co-cultured with WT or MPN (ET-like or PV-like) senescent neutrophils treated with anti-CD24 blocking antibody or control IgG (created in BioRender.com).

(A, C-H) Data are means±SEM. Each dot is a mouse.

(A, C-F) Student’s two-tailed unpaired t test.

(G-H) Two-way ANOVA and pairwise comparisons.

**Supplementary Figure 2. High CD24 expression in *JAK2^V617F+^* neutrophils.**

(A) Flow cytometry histogram showing the geometric mean of CD24 expression in human blood neutrophils from MPN patients and healthy controls (HC).

(B) CD24 protein expression in BM hematopoietic cells from WT mice or essential thrombocythemia (ET)-like mice (n=3-7). Neut, CD11b^+^ SSC^hi^ neutrophils; Mac, CD11b^+^ F4/80^+^ macrophages; MK, FSC^hi^ CD11b^-^ CD41^+^ megakaryocytes; PLT, FSC^low^ CD11b^-^ CD41^+^ platelets; NK, FSC^+^/SSC^+^NK1.1^+^ natural killer cells.

(C) CD24 protein expression in BM hematopoietic stem cells (SLAM LSK; Lin^-^/Sca-1^+^/c-kit^+^/CD48^-^/CD150^+^) from WT or MPN mice (n=4-5).

(D-E) CD24 protein expression in blood (D) neutrophils (healthy controls; n=14, MPNs- JAK2^V617F^; n=23; duplicate from Figure 1I), (E) hematopoietic progenitor cells (CD34^+^CD38^+^) or hematopoietic stem cells (CD34^+^CD38^-^) cells from healthy controls (n=6) and MPN JAK2^V617F+^ patients (n=3).

(F) Siglec-G protein expression in BM CD11b^+^ F4/80^+^ macrophages from WT (n=4) or ET-like (n=4) mice. ET mice were analyzed at early stage of disease (age 13-16w).

(G-H) Representative examples of labelled human MPN granulocytes (green) co-cultured with macrophages in presence of anti-CD24 blocking antibody or control IgG. Arrowheads depict phagocytic macrophages. Scale bar, 20μm.

(I) Flow cytometry plots and quantification of WT macrophages phagocytosing MPN neutrophils treated with anti-CD24, anti-CD47 or control IgG antibodies (n=4-5).

(J) Experimental workflow of adoptive transfer. WT mice were injected with WT or *JAK2^V617F^*DsRed^+^ neutrophils (i.v.) and anti-CD24 blocking antibody or control IgG (i.p.) and were sacrificed 24h later (created in BioRender.com).

(K) *CFS2RA* mRNA expression in blood granulocytes from MPN patients including essential thrombocythemia (ET, n=25), polycythemia vera (PV, n=23) and primary myelofibrosis (PMF, n=7), compared with healthy controls (n=8)^14^.

(L) CD24 expression in senescent neutrophils from MPN mice after 16h culture with GM-CSF (n=7), STAT3 inhibitor (n=5) or vehicle (n=7).

(M-O) Quantification (M) and flow cytometry histogram (N) of CD24 expression in senescent neutrophils isolated from WT mice or *Mx1-Cre*;*Stat5^fl/fl^* mice (induced with pIpC), after 16h culture with GM-CSF (n=4-5 per group).

(O) Expression of CD47 in senescent BM neutrophils from WT or induced *Mx1-Cre*;*Stat5^fl/fl^* mice after 16h culture with GM-CSF (n=4-5 per group).

(B-F, I, K-L, M, O) Data are means±SEM. Each dot is a mouse or individual.

(C-F) Student’s two-tailed unpaired t test.

(B, M, O) Two-way ANOVA and pairwise comparisons.

(I, K, L) One-way ANOVA and pairwise comparisons.

**Supplementary Figure 3. Reduced concentration of GM-CSF and other inflammatory cytokines, in BM of myeloid-specific (*LysM-Cre;JAK2^V617F^*) mice, compared with pan-hematopoietic (*Vav-Cre;JAK2^V617F^*) mice.**

**(**A-E) Inflammatory cytokines (IL-4, IL-6, IL-7, IL-10, IL-17 and GM-CSF) concentration in the BM of *LysM-Cre;JAK2^V617F^* mice, compared with ***Vav-Cre;JAK2^V617F^*** MPN mice (n=3-10 per group).

(F-G) Frequency of neutrophils (F) or senescent neutrophils (G) in the BM of *LysM-Cre;JAK2^V617F^* mice (n=6) or WT mice (n=5-8).

(H-I) CD24 expression in (H) senescent neutrophils freshly isolated from *LysM-Cre;JAK2^V617F^* mice or WT mice, or (I) analyzed 16h after incubation with GM-CSF or vehicle (n=4).

(A-I) Data are means±SEM. Each dot is a mouse. Student’s two-tailed unpaired t test.

**Supplementary Figure 4. Pathogenic interactions of neutrophils with megakaryocytes in MPN.**

(A-B) Representative examples of BM CD41^+^ (green) megakaryocytes and Ly6G^+^ (red) neutrophils from (A) WT or (B) polycythemia vera (PV)-like mice. Scale bar, 50µm.

(C-D) Representative examples (C) and quantification (D) of *in vitro* emperipolesis of WT megakaryocytes co-cultured with WT or *JAK2^V617F^* neutrophils (n=5-6 per group). Scale bar, 100μm.

(E) Experimental workflow for *in vitro* emperipolesis assay of WT megakaryocytes with freshly isolated CD62L^high^ young neutrophils or CD62L^low^ senescent neutrophils isolated from *JAK2^V617^*^F^ *β-actin-DsRed* mice (created in BioRender.com).

(F-G) Representative examples (F) and quantification (G) of *in vitro* emperipolesis of *VWF*-eGFP^+^ (green) megakaryocytes with DsRed^+^ (red) CD62L^high^ young or CD62L^low^ senescent neutrophils (n=3). Scale bar, 250µm.

(D, G) Data are means±SEM. Each dot is a mouse. Student’s two-tailed unpaired t test.

**Supplementary Figure 5. Megakaryocyte emperipolesis of neutrophils increases platelet release.**

(A) Scheme of functionalized 3D scaffold-perfused bioreactor to evaluate human proplatelet formation and emperipolesis, and to measure thrombopoiesis in real-time. A representative flow cytometry plot of platelets collected from the bioreactor system is shown (black: platelets; pink: counting beads) (created in BioRender.com).

(B) Representative example of deepRed^+^ neutrophils (red) soon after seeding into the 3D silk BM model. Scale bar, 100 µm.

(C-D) Representative example of human CD61^+^ (green) megakaryocytes and CD66b^+^ (red) neutrophils from MPN patients in the silk BM model. Scale bar, 20µm (C), 50µm (D).

(E-F) Representative examples of proplatelet-formation form megakaryocytes (E) cultured alone or (F) co-cultured with elastase^+^ (green) neutrophils from healthy donor. Scale bar, 50µm (E), 20µm (F).

(G) Quantification of platelet release in the 3D model under perfusion. Data are means±SEM. Each dot is an individual. Student’s two-tailed unpaired t test.

(H-I) Snapshot of live-imaging from CFSE^+^ (green) human megakaryocytes and Deep-Red^+^ (red) neutrophils derived from healthy donors and cultured in the silk BM model under perfusion. Release of platelet-like particles is evidenced under perfusion from the collected flow. Scale bar 100µm.

(J) Experimental workflow of repeated adoptive transfer of WT or *JAK2^V617^*^F^ senescent neutrophils (created in BioRender.com).

(K) Circulating platelet counts after repeated adoptive transfer of WT senescent neutrophils into WT mice or from untreated WT mice (n=4-9 per group). Each dot is the mean of each group.

**Supplementary Figure 6. CD24 deletion or blockade does not cause hematological toxicity in MPN mouse models.**

(A) Representative flow cytometry histogram showing the geometric mean of CD24 expression in BM senescent neutrophils from WT mice, control mouse model of essential thrombocythemia (ET-*Cd24^+/+^)* or *Vav1-Cre*;*JAK2^V617F^* mice backcrossed to *Cd24^-/-^* mice to delete CD24 (ET-*Cd24^-/-^* mice).

(B) Experimental workflow of non-competitive transplantation of BM cells from ET *Cd24^+/+^* or ET *Cd24^-/-^* mice into WT recipients (created in BioRender.com).

(C) Experimental workflow of chronic treatment of ET-like or polycythemia vera (PV)-like MPN mice with anti-CD24 blocking antibody (clone M1/69) or control rat IgG2b (created in BioRender.com).

(D-H) Peripheral blood counts in *Cd24^-/-^* mice or WT mice (n=4-5).

(I-M) Peripheral blood counts (I; white blood cells (WBCs), J; red blood cells (RBCs), K; hematocrit (HCT) and L; hemoglobin (HGB)) and (M) body weight of PV-like mice treated for 11 weeks with anti-CD24 blocking antibody or isotype control (n=6-8).

(N) Peripheral blood chimerism of mutant PV CD45.2 from PV-like mice treated for 8 weeks with anti-CD24 blocking antibody or isotype control (n=4-6).

(O-S) Peripheral blood counts (O; white blood cells (WBCs), P; red blood cells (RBCs), Q; hematocrit (HCT) and R; hemoglobin (HGB)) and (S) body weight of ET-like mice treated for 11 weeks with anti-CD24 blocking antibody or isotype control (n=6-7).

(D-S) Data are means±SEM. Each dot is a mouse.

(D-H) Student’s two-tailed unpaired t test.

(I-S) Two-way ANOVA and pairwise comparisons.

**Supplementary Figure 7. CD24 deletion or blockade prevents megakaryocyte emperipolesis of neutrophils and improves thrombocytosis in MPN.**

(A-B) Analysis of MPN hematopoietic chimeras proficient for CD24 (ET *Cd24^+/+^*) or lacking CD24 in hematopoietic cells only (ET *Cd24^-/-^*). Frequency of (A) blood or (B) BM CD11b^+^ Ly6G^+^ neutrophils (n=5-7).

(C) Flow cytometry plots of BM CD11b^+^ Ly6G^+^ CD62L^lo^ senescent neutrophils from chimeric ET mice proficient/deficient for CD24 expression in hematopoietic cells.

(D) Frequency of BM senescent neutrophils from WT mice treated with anti-CD24 blocking antibody (clone M1/69) or control rat IgG2b for 7w (n=3-4).

(E) Quantification of *in vitro* emperipolesis of WT megakaryocytes with MPN neutrophils treated with anti-CD24 or anti-P-selectin (RB40.34) blocking antibodies, or control IgG (n=3-6).

(F) Platelets recovered from perfused bioreactor containing human megakaryocytes derived from healthy donors treated with anti-CD24 blocking antibody (clone SN3), or control mouse IgG1 (n=3).

(G) Immunofluorescence of human megakaryocytes and proplatelet formation (beta1-Tubulin; green, Nuclei-DAPI; blue) treated with anti-CD24-blocking antibody (clone SN3) or control mouse IgG1. Scale bar, 20 µm.

(H) Circulating platelets (PLT) counts of *Cd24^-/-^* mice or WT mice (n=4-5 per group).

(I) Circulating platelets (PLT) counts of WT mice treated over 7w with anti-CD24 blocking antibody or control IgG (n=4).

(A-B, D-F, H-I) Data are mean±SEM. Each dot is a mouse or individual.

(A-B, D, F, H) Student’s two-tailed unpaired t test.

(E) One-way ANOVA and pairwise comparisons.

(I) Two-way ANOVA and pairwise comparisons.

**Supplementary Figure 8. Neutrophil degranulation is associated with increased active TGF-β and myelofibrosis.**

(A-B) Supervised analysis of human MPN granulocyte RNAseq^16^ showing weak correlation between CD47 expression and neutrophil degranulation signatures.

(C) Model of neutrophil degranulation and proteolytic enzymes leading to activation of latent pro-fibrotic TGF-β.

(D) Experimental workflow of measuring active TGF-b in the 3D silk BM model.

(E-F) Representative examples of CD61^+^ (green) megakaryocytes and elastase^+^ (red) neutrophils from (E) control healthy donors or (F) MPN patients. Scale bar, 20μm.

(G) Active TGF-β concentration in central BM of WT or ET-like mice (n=7-9). Data are mean±SEM. Each dot is a mouse. Student’s two-tailed unpaired t test.

(H) Gömöri staining of reticulin fibrosis in BM section from ET-like mice treated with anti-CD24 blocking antibody or control IgG for 11w. Scale bar, 50μm.

**Supplementary Table 1. List of human samples and clinical characteristics of MPN patients.**

**Supplementary Table 2. Supervised analyses of human granulocyte microarray datasets.**

Data analyzed from MPN cases of polycythemia vera (PV, n=28), essential thrombocythemia (ET, n=47) and myelofibrosis (MF, n=18), and age-matched healthy donors (n=11)^14^. Log2FC values >0 indicates upregulated genes.

**Supplementary References**

1.	Carrelha J, Meng Y, Kettyle LM, et al. Hierarchically related lineage-restricted fates of multipotent haematopoietic stem cells. *Nature*. 2018;554(7690):106-111.

2.	Tiedt R, Hao-Shen H, Sobas MA, et al. Ratio of mutant JAK2-V617F to wild-type Jak2 determines the MPD phenotypes in transgenic mice. *Blood*. 2008;111(8):3931-3940.

3.	Li J, Prins D, Park HJ, et al. Mutant calreticulin knockin mice develop thrombocytosis and myelofibrosis without a stem cell self-renewal advantage. *Blood*. 2018;131(6):649-661.

4.	Cui Y, Riedlinger G, Miyoshi K, et al. Inactivation of Stat5 in mouse mammary epithelium during pregnancy reveals distinct functions in cell proliferation, survival, and differentiation. *Mol Cell Biol*. 2004;24(18):8037-8047.

5.	Grockowiak E, Korn C, Rak J, et al. Different niches for stem cells carrying the same oncogenic driver affect pathogenesis and therapy response in myeloproliferative neoplasms. *Nat Cancer*. 2023;4(8):1193-1209.

6.	Furze RC, Rankin SM. Neutrophil mobilization and clearance in the bone marrow. *Immunology*. 2008;125(3):281-288.

7.	Bench AJ, Baxter EJ, Green AR. Methods for detecting mutations in the human JAK2 gene. *Methods Mol Biol*. 2013;967:115-131.

8.	Ho YH, Del Toro R, Rivera-Torres J, et al. Remodeling of Bone Marrow Hematopoietic Stem Cell Niches Promotes Myeloid Cell Expansion during Premature or Physiological Aging. *Cell Stem Cell*. 2019;25(3):407-418.

9.	Di Buduo CA, Alberelli MA, Glembostky AC, et al. Abnormal proplatelet formation and emperipolesis in cultured human megakaryocytes from gray platelet syndrome patients. *Sci Rep*. 2016;6:23213.

10.	Di Buduo CA, Soprano PM, Miguel CP, Perotti C, Del Fante C, Balduini A. A Gold Standard Protocol for Human Megakaryocyte Culture Based on the Analysis of 1,500 Umbilical Cord Blood Samples. *Thromb Haemost*. 2021;121(4):538-542.

11.	Abbonante V, Di Buduo CA, Gruppi C, et al. Thrombopoietin/TGF-beta1 Loop Regulates Megakaryocyte Extracellular Matrix Component Synthesis. *Stem Cells*. 2016;34(4):1123-1133.

12.	Di Buduo CA, Soprano PM, Tozzi L, et al. Modular flow chamber for engineering bone marrow architecture and function. *Biomaterials*. 2017;146:60-71.

13.	Isern J, Garca-Garca A, Martn AM, et al. The neural crest is a source of mesenchymal stem cells with specialized hematopoietic stem-cell-niche function. *eLife*. 2014;3.

14.	Rampal R, Al-Shahrour F, Abdel-Wahab O, et al. Integrated genomic analysis illustrates the central role of JAK-STAT pathway activation in myeloproliferative neoplasm pathogenesis. *Blood*. 2014;123(22):e123-133.

15.	Ritchie ME, Phipson B, Wu D, et al. limma powers differential expression analyses for RNA-sequencing and microarray studies. *Nucleic Acids Res*. 2015;43(7):e47.

16.	Schischlik F, Jager R, Rosebrock F, et al. Mutational landscape of the transcriptome offers putative targets for immunotherapy of myeloproliferative neoplasms. *Blood*. 2019;134(2):199-210.

17.	Kim D, Paggi JM, Park C, Bennett C, Salzberg SL. Graph-based genome alignment and genotyping with HISAT2 and HISAT-genotype. *Nat Biotechnol*. 2019;37(8):907-915.

18.	Love MI, Huber W, Anders S. Moderated estimation of fold change and dispersion for RNA-seq data with DESeq2. *Genome Biol*. 2014;15(12):550.

19.	Subramanian A, Tamayo P, Mootha VK, et al. Gene set enrichment analysis: a knowledge-based approach for interpreting genome-wide expression profiles. *Proc Natl Acad Sci U S A*. 2005;102(43):15545-15550.

20.	Hanzelmann S, Castelo R, Guinney J. GSVA: gene set variation analysis for microarray and RNA-seq data. *BMC Bioinformatics*. 2013;14:7.
